# Structure and Assembly Pathway of the Ribosome Quality Control Complex

**DOI:** 10.1016/j.molcel.2014.12.015

**Published:** 2015-02-05

**Authors:** Sichen Shao, Alan Brown, Balaji Santhanam, Ramanujan S. Hegde

**Affiliations:** 1 MRC Laboratory of Molecular Biology, Francis Crick Avenue, Cambridge, CB2 0QH, UK

## Abstract

During ribosome-associated quality control, stalled ribosomes are split into subunits and the 60S-housed nascent polypeptides are poly-ubiquitinated by Listerin. How this low-abundance ubiquitin ligase targets rare stall-generated 60S among numerous empty 60S is unknown. Here, we show that Listerin specificity for nascent chain-60S complexes depends on nuclear export mediator factor (NEMF). The 3.6 Å cryo-EM structure of a nascent chain-containing 60S-Listerin-NEMF complex revealed that NEMF makes multiple simultaneous contacts with 60S and peptidyl-tRNA to sense nascent chain occupancy. Structural and mutational analyses showed that ribosome-bound NEMF recruits and stabilizes Listerin’s N-terminal domain, while Listerin’s C-terminal RWD domain directly contacts the ribosome to position the adjacent ligase domain near the nascent polypeptide exit tunnel. Thus, highly specific nascent chain targeting by Listerin is imparted by the avidity gained from a multivalent network of context-specific individually weak interactions, highlighting a new principle of client recognition during protein quality control.

## Highlights

• Targeting of Listerin E3 ligase to translationally stalled proteins requires NEMF
• NEMF binds to the 60S subunit, prevents 40S rejoining, and stabilizes Listerin
• Cryo-EM structure of the 60S-NEMF-Listerin complex reveals a network of interactions
• NEMF discriminates 60S-nascent chains from empty 60S via exposed peptidyl tRNA

## Graphical Abstract

**Figure:**
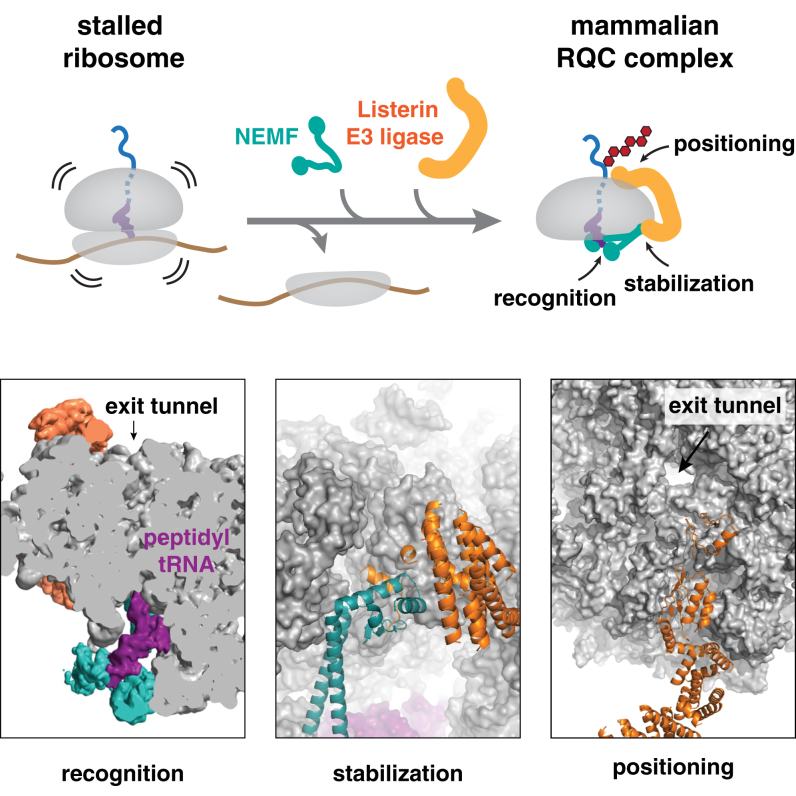

## Teaser

Ribosomes that stall during translation are dissociated, and the aberrant polypeptide within the 60S ribosomal subunit is poly-ubiquitinated by Listerin. Shao et al. identify NEMF as a factor that facilitates Listerin recruitment to 60S-nascent chains and provide structural insights into how targets are selected during this ribosome-associated quality control pathway.

## Introduction

Quality control is a pervasive aspect of every biosynthetic process ranging from DNA replication and transcription, to mRNA translation, protein folding, and subcellular localization (Rodrigo-Brenni and Hegde, 2012; Wolff et al., 2014). Failure of any of these quality control pathways is invariably detrimental to cellular fitness and is the basis for a wide range of human diseases. To be effective, quality control pathways must be tuned appropriately to maximize the targeting of aberrant products while minimizing engagement of normal counterparts. Thus, a central issue for any quality control pathway is the mechanistic basis of high-fidelity target selection.

During protein quality control, aberrant polypeptides are typically marked for degradation by ubiquitin ligases that must be preferentially targeted to their clients. Accurate identification of aberrant proteins poses several challenges to the cell including their high similarity to normal biosynthetic intermediates, the need to accommodate a diverse client range, and their relative rarity under normal conditions. The precise features that are recognized to identify an aberrant protein and the mechanistic basis of their accurate recognition are poorly understood for most protein quality control pathways.

One of the earliest points of protein quality control is a ribosome-associated pathway for stalled translation products. Ribosomes can stall during translation elongation for a number of reasons, each of which triggers protein and mRNA quality control pathways (Lykke-Andersen and Bennett, 2014; Shoemaker and Green, 2012). The two main pathways, first recognized in the context of mRNA degradation, are no-go decay and nonstop decay. No-go decay occurs when translation halts within an open reading frame (due to mRNA truncation, secondary structure, or rare codons), while nonstop decay occurs when ribosomes read into and stall within the poly(A) tail (Doma and Parker, 2006; Frischmeyer et al., 2002; van Hoof et al., 2002). The protein products of these translational stalls are degraded via the ubiquitin-proteasome system (Dimitrova et al., 2009). While some stalled polypeptides may be prematurely terminated (Chiabudini et al., 2014), a predominant pathway for their ubiquitination occurs at the ribosome (Bengtson and Joazeiro, 2010), committing them to degradation before release into the bulk cytosol.

In yeast, nascent chain ubiquitination requires the ubiquitin ligase Ltn1 (Bengtson and Joazeiro, 2010; Brandman et al., 2012; Defenouillère et al., 2013). In vitro analysis in lysate-based and purified reconstituted systems showed that its mammalian homolog Listerin was both necessary and sufficient for ubiquitination of stalled translation products (Shao and Hegde, 2014; Shao et al., 2013). Nascent chain ubiquitination in these studies required splitting of the 80S ribosome-nascent chain (RNC) into subunits by the ribosome recycling factors Pelota, Hbs1, and ABCE1 (Shao and Hegde, 2014; Shao et al., 2013). While very short peptidyl-tRNAs drop off the ribosome upon splitting (Pisareva et al., 2011; Shao et al., 2013; Shoemaker et al., 2010), longer polypeptide-tRNAs remain within the 60S subunit to generate 60S-RNCs (Shao et al., 2013). Ubiquitination assays with isolated 60S- versus 80S-RNCs showed that Listerin strongly favors the former complex (Shao and Hegde, 2014). These observations, together with cofractionation of Listerin with 60S-RNCs, argue that Listerin accesses stalled RNCs only after 40S subunit removal. This model is consistent with studies in yeast showing Ltn1 copurification with 60S (Bengtson and Joazeiro, 2010; Brandman et al., 2012; Defenouillère et al., 2013) and stabilization of nascent chains in strains lacking the Pelota homolog Dom34 (Izawa et al., 2012; Verma et al., 2013).

In addition to Ltn1 and ribosome splitting factors, genetic studies in yeast have identified additional components in the ribosome-associated quality control (RQC) pathway. The ribosome-associated proteins Asc1 (Brandman et al., 2012; Kuroha et al., 2010) and Hel2 (Brandman et al., 2012) facilitate stalling at poly-basic residues, such as lysines encoded by poly(A) tails. After stalling, genes needed for efficient nascent chain degradation include Rqc1, Tae2, and the Cdc48 complex (Brandman et al., 2012; Defenouillère et al., 2013; Verma et al., 2013). All of these components were isolated together with 60S and Ltn1 (Brandman et al., 2012; Defenouillère et al., 2013), defining an RQC complex.

Structural analyses of the RQC complex by electron cryo-microscopy (cryo-EM) have begun to reveal its overall architecture. The structure of a functional Listerin-60S-RNC complex reconstituted from purified components showed that Listerin’s position clashes with 40S, explaining why it prefers 60S over 80S (Shao and Hegde, 2014). Ltn1 was observed in a similar position in the cryo-EM reconstruction of a native RQC complex affinity purified from yeast (Lyumkis et al., 2014). This structure contained additional density that appeared to bridge Ltn1 and a tRNA bound at the P-site. Absence of the bridging density in reconstructions of the RQC complex isolated from *Tae2Δ* yeast suggested the intriguing possibility that Tae2 may sense 60S occupancy via P-site tRNA (Lyumkis et al., 2014). However, it was not possible to unambiguously assign Tae2 or resolve its interactions in this moderate-resolution structure. Furthermore, the biochemical function of Tae2 remains uncertain, as *TAE2* deletion has yielded contradictory effects on Ltn1 association with 60S RNCs (Brandman et al., 2012; Defenouillère et al., 2013; Lyumkis et al., 2014). Thus, the assembly pathway and molecular basis for high-fidelity target selection by the RQC complex remain poorly understood.

Here, we show that Listerin acts in concert with NEMF (the mammalian Tae2 homolog) to select its targets. NEMF functions by sensing nascent chain occupancy of 60S, preventing subunit reassociation, and stabilizing Listerin on 60S. Structural analysis provided a mechanistic explanation for each of these three activities by revealing the network of interactions made by 60S-RNCs with NEMF and Listerin. An exposed P-site tRNA was the decisive feature used by NEMF to discriminate between occupied and empty 60S. The aberrancy of 60S-RNCs is therefore recognized by the juxtaposition of two normal elements, a tRNA and the 60S ribosomal subunit, neither of which is individually sufficient. Thus, the avidity from multiple context-dependent interactions is exploited for quality control recognition in the RQC pathway.

## Results and Discussion

### NEMF Facilitates Listerin Recruitment to Stalled RNCs

**Figure 1:**
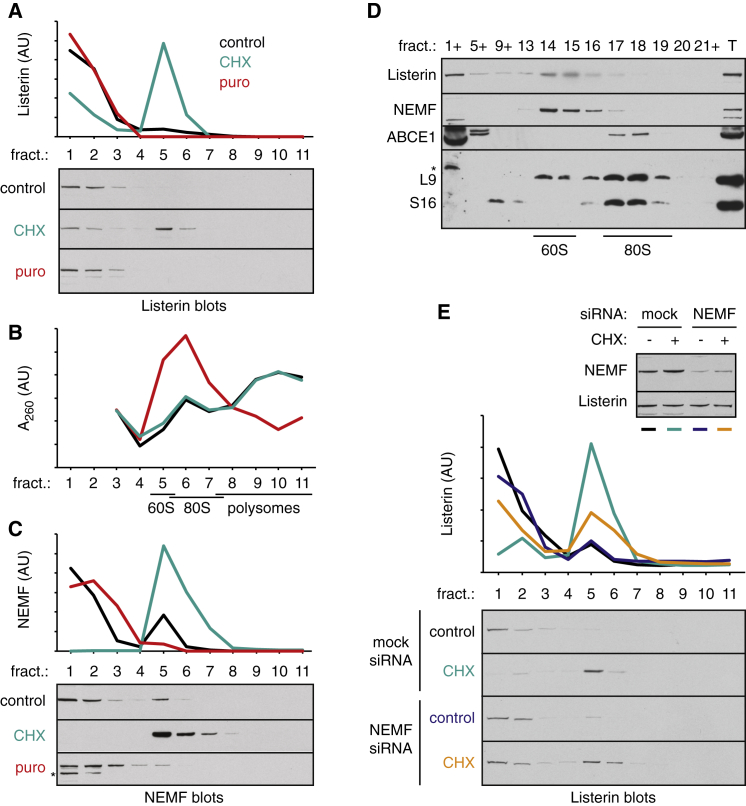
NEMF Is Required for Listerin Recruitment to Stalled Ribosomes (A) HEK293T cells treated for 30 min with DMSO (control), 50 μg/ml CHX, or 1 mM puromycin (puro) were lysed and the cytosolic extracts separated on 10%–50% sucrose gradients. Eleven fractions from top (fraction 1) to bottom (fraction 11) were analyzed for the distribution of Listerin. The immunoblots and their respective quantification are shown. (B) The A_260_ profiles of the sucrose gradients from (A) are shown. As shown in Figure S1A, fractions 5 corresponds to 60S, fraction 6 to 80S, and fractions 7–11 to polysomes. (C) Immunoblotting of the samples from (A) for NEMF recruitment to ribosomes. (D) Cytosolic extract from CHX-treated cells as in (A) were size fractionated on a high-resolution 10%–30% sucrose gradient and immunoblotted for the indicated components. The positions of 60S and 80S are indicated. L9 (uL6) and S16 (uS9) are small and large subunit proteins, respectively. T indicates total lysate. Asterisk indicates background band. The plus symbol indicates multiple fractions were pooled (e.g., 5+ is fractions 5–8). (E) HEK293T cells transfected for 30 hr with mock or NEMF siRNAs were analyzed for Listerin recruitment to ribosomes as in (A). The inset shows Listerin and NEMF levels in the total cytosol extract. Knockdown of NEMF was to ∼25% of control levels. See also Figure S1.

In a purified reconstituted system, Listerin alone is sufficient to ubiquitinate 60S-RNCs after splitting of 80S-RNCs (Shao and Hegde, 2014). In a physiologic setting however, 60S-RNCs would be rare relative to 60S generated by the recycling of empty ribosomes. To determine if Listerin has strong specificity for nascent chain-containing 60S in vivo, we used translation inhibitors to generate 60S subunits containing or lacking nascent chains. Cycloheximide (CHX) stalls ribosomes during elongation, with ribosome splitting factors converting at least some of the RNCs into nascent chain-containing 60S subunits (Shao et al., 2013). Puromycin also interrupts translation elongation, but by premature chain release; this results in 80S ribosomes that are recycled by splitting factors to generate empty 60S subunits (Pisareva et al., 2011). We found that while Listerin was efficiently recruited to the 60S fraction upon CHX treatment, no recruitment was observed with puromycin (Figure 1A), despite these cells containing several-fold more 60S subunits (as judged by A_260_; Figures 1B and S1A). Thus, in vivo, Listerin displays high target specificity for occupied 60S over the more abundant polysomes, 80S ribosomes, and empty 60S subunits.

However, Listerin was not stably associated with ribosomes isolated from ubiquitination reactions reconstituted with purified factors (Figure S1B available online). The 60S-RNC was itself a transient species; it was efficiently converted to 80S via subunit reassociation (Figure S1C) unless excess eIF6 was present to bind the intersubunit interface (Gartmann et al., 2010; Shao and Hegde, 2014). This indicates that while Listerin can dynamically associate with 60S-RNCs for sufficiently long to poly-ubiquitinate the nascent chain (Shao and Hegde, 2014), Listerin binding and subunit reassociation are strong competing reactions in the purified reconstituted system. By contrast, Listerin is stably bound to 60S-RNCs in cells or lysate-based in vitro reactions (Bengtson and Joazeiro, 2010; Brandman et al., 2012; Defenouillère et al., 2013; Shao et al., 2013), suggesting that the purified system lacks factor(s) that stabilize the Listerin-60S complex.

Candidates for this factor should be found in native 60S-RNC complexes together with Listerin. Analysis of the 60S RQC complex in yeast had revealed Rqc1, Tae2, Asc1, and the Cdc48 complex as abundant constituents (Brandman et al., 2012; Defenouillère et al., 2013). Of these, the Tae2 homolog NEMF was the highest hit (comparable to Listerin) in our mass spectrometry analysis of native mammalian 60S-RNC complexes (Shao and Hegde, 2014). By contrast, the Rqc1 homolog was not detected, the Asc1 homolog associates with 40S, and the Cdc48 homolog was not selective to nascent chain-containing complexes.

NEMF is poorly characterized in metazoans, having been linked to colon and lung cancers and implicated indirectly in mediating nuclear export (Bi et al., 2005; Carbonnelle et al., 1999). Studies in yeast clearly implicate Tae2 in non-stop protein degradation (Brandman et al., 2012; Defenouillère et al., 2013), and the recent cryo-EM reconstruction of native yeast RQC complex suggests that Tae2 binds both Ltn1 and P-site tRNA (Lyumkis et al., 2014). However, its mechanistic role in the RQC pathway remains poorly characterized. While one study found impaired Ltn1 association with 60S subunits in *Tae2Δ* yeast (Defenouillère et al., 2013), other studies could efficiently recover Ltn1-60S complexes without Tae2 (Brandman et al., 2012; Lyumkis et al., 2014), instead implicating a role for Tae2 in signaling heat shock stress (Brandman et al., 2012).

Given its strong genetic and physical interactions with Listerin, we examined NEMF’s function. We observed that acute treatment of cultured cells with CHX selectively converted most cellular NEMF from a soluble to a ribosome-associated state, mirroring Listerin (Figure 1C). By contrast, neither puromycin nor pactamycin (a translation initiation inhibitor) stimulated NEMF recruitment to ribosomes (Figure 1C; data not shown). Higher resolution sucrose gradients confirmed that both NEMF and Listerin were recruited to 60S subunits, while the splitting factor ABCE1 was on 80S (Figure 1D). Importantly, siRNA knockdown of NEMF to ∼25% (Figure S1D) resulted in diminished recruitment of Listerin to stalled ribosomes (Figure 1E), suggesting that NEMF facilitates Listerin recruitment to mammalian RQC substrates.

### NEMF Is Necessary and Sufficient for Listerin Recruitment to 60S-RNCs

**Figure 2:**
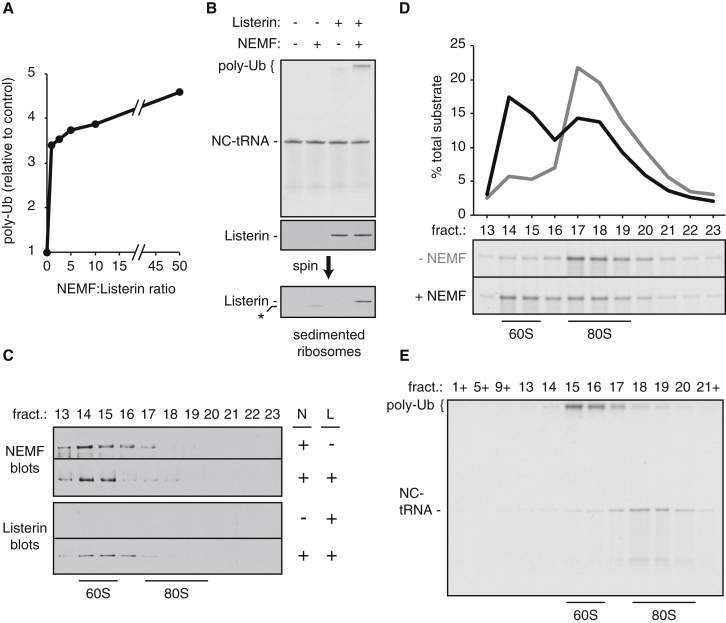
NEMF Is Sufficient to Stabilize Listerin on 60S RQC Complexes (A) Purified NEMF was titrated into ubiquitination reactions containing 5 nM affinity-purified ^35^S-labeled stalled 80S-RNCs and 1.2 nM Listerin. All reactions contained splitting factors (50 nM Hbs1, 50 nM Pelota, and 50 nM ABCE1), ubiquitination reagents (75 nM E1, 250 nM UbcH5, and 10 μM ubiquitin), and an energy regeneration system. Reactions were incubated at 32°C for 10 min before analysis by SDS-PAGE and autoradiography. The amount of poly-ubiquitination was quantified and normalized to that seen without NEMF (set arbitrarily to 1). (B) Radiolabeled 80S-RNCs were subjected to ubiquitination reactions containing or lacking NEMF and Listerin as indicated. Reactions were at 32°C for 3 min. Reaction products were analyzed by SDS-PAGE and autoradiography. The tRNA-attached nascent chain (NC-tRNA) and its poly-ubiquitinated species (poly-Ub) are indicated. A parallel aliquot of the reaction was separated by centrifugation to isolate ribosomes. Listerin was detected by immunoblot in the total (middle panel) and ribosome fraction (bottom panel). Asterisk indicates position of a background band. (C) Reactions as in (B) were separated on 10%–30% sucrose gradients and the indicated fractions analyzed by SDS-PAGE and immunoblotting for NEMF or Listerin. The fractions corresponding to 60S and 80S are indicated. (D) 80S-RNCs were incubated with splitting factors and energy without (gray) or with (black) 50 nM NEMF and separated on 10%–30% sucrose gradients. The profiles of the NC-tRNA are displayed. Note that Listerin was not included in these reactions so that NC-tRNA could be visualized as a single band. (E) Ubiquitination reaction of radiolabeled 80S-RNCs with 1.2 nM Listerin and 1.2 nM NEMF were separated on a 10%–30% sucrose gradient and analyzed by autoradiography. Unmodified NC-tRNA, its poly-ubiquitinated species, and the positions of 60S and 80S are indicated. Note that essentially all 60S-RNCs are poly-ubiquitinated, while 80S-RNCs remain largely, if not completely, unmodified. See also Figure S2.

To examine this hypothesis rigorously, we analyzed NEMF in our recently characterized purified reconstituted system for RNC ubiquitination (Shao and Hegde, 2014). Recombinant NEMF purified from HEK293T cells (Figure S2A) was titrated into a reaction containing ^35^S-labeled 80S-RNCs, ribosome splitting factors, Listerin, E1 and E2 enzymes, ubiquitin, and energy. Ubiquitination of the radiolabeled nascent chain-tRNA substrate, monitored by the production of a heterogeneous high-molecular-weight product, was ∼3- to 5-fold higher with NEMF addition (Figure 2A). NEMF alone had no ubiquitination activity (Figure 2B), and its stimulatory effect reached near-maximal amounts at concentrations equimolar to Listerin. Ubiquitination in the presence of NEMF was rapid (Figure S2B) and more processive than with Listerin alone (Figure S2C). Isolation of ribosomal complexes from these samples revealed Listerin recruitment preferentially in reactions containing NEMF (Figure 2B), consistent with the results in cells (Figure 1C). Thus, NEMF stimulates Listerin-mediated ubiquitination and stabilizes the Listerin-ribosome interaction.

NEMF associates with 60S-RNCs even in the absence of Listerin (Figure 2C, top panel); Listerin binding under identical conditions depends on NEMF (Figure 2C, bottom panel), even though its transient interaction with 60S-RNCs could be inferred from the observed ubiquitination (Figure 2B). This transient versus stable interaction presumably explains the increased processivity of ubiquitination seen with NEMF (Figure S2C). When added in excess of RNCs, NEMF together with splitting factors was sufficient to redistribute most RNCs from the 80S to 60S fractions (Figure 2D), suggesting that NEMF binding to 60S-RNCs effectively precludes 40S reassociation. Importantly, ubiquitinated nascent chains migrate exclusively in 60S fractions when NEMF is present (Figure 2E), whereas 40S reassociation into 80S complexes was prevalent in its absence (Figure S1C). Taken with our observations in mammalian cells, these results indicate that NEMF is both necessary and sufficient for stable Listerin association with 60S-RNCs. NEMF appears to be recruited to 60S-RNCs first, where it prevents 40S subunit reassociation and stabilizes the Listerin-60S interaction to enhance nascent chain ubiquitination.

### Architecture of 60S-RNCs Bound to NEMF and Listerin

To understand the mechanistic basis of NEMF’s functions, we turned to single-particle reconstruction of this complex using cryo-EM. Purified stalled 80S-RNCs were incubated with ribosome splitting factors, NEMF, Listerin, and energy to produce ubiquitination-competent 60S-RNCs (Figure S3A). The resulting complexes were analyzed by cryo-EM leading to the identification and initial interpretation of the major structural features. A later data set was collected on a similar specimen that also included TCF25 (the mammalian Rqc1 homolog) in the reaction. However, the resulting reconstruction was indistinguishable from the initial data set, and biochemical experiments showed that TCF25 neither associated with 60S-RNCs nor stimulated ubiquitination (data not shown). We therefore combined the two data sets to maximize the resolution of the reconstructed map (Figure S3B).

**Figure 3:**
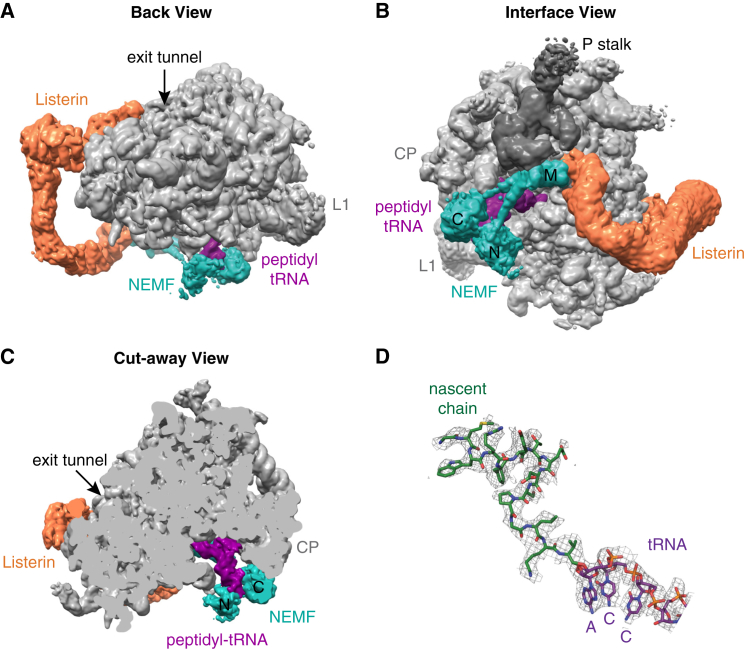
Architecture of 60S-RNCs Bound to NEMF and Listerin (A) Back view of a cryo-EM reconstruction of the 60S-RNC complex showing the ribosome in gray, Listerin in orange, NEMF in teal, and the P-site peptidyl-tRNA in purple. The nascent polypeptide exit tunnel and the ribosomal L1 stalk are indicated for orientation. The map is low-pass filtered to 5 Å and displayed at a threshold to visualize continuous density of the factors. (B) View from the subunit interface of the 60S-RNC complex. The central protuberance (CP) and L1 stalk are shown for orientation. The P stalk of the ribosome is in dark gray. The N-terminal lobe (N), C-terminal lobe (C), and Middle domain (M) of NEMF are labeled. (C) Cut-away view of the 60S-RNC complex showing that the N- and C-lobes of NEMF contact the tRNA and a region of Listerin poised at the exit tunnel. (D) Example of the fit of a docked tRNA (purple), showing the 3′ CCA end, and the de novo model of the defined nascent chain (green) into the EM map density. See also Figure S3.

The combined data set containing 117,461 particles was processed through RELION (Scheres, 2012). After initial 3D refinement, movie processing was performed to adjust for drift and radiation damage (Bai et al., 2013), resulting in an initial map that showed extraribosomal density we provisionally assigned to Listerin and NEMF. These particles were then subjected to further 3D classification with a mask around the presumed Listerin and NEMF densities to enrich for their occupancy. The enriched class, containing 63,826 particles, was refined (Table 1) to produce our final map of a 60S-RNC in complex with Listerin and NEMF (Figure 3). Gold standard FSC curve analysis indicated an overall resolution of 3.6 Å (Figure S3C). The ribosome displayed the highest resolution, while local resolution for the associated factors decreased relative to the core of the ribosome (Figure S3D).

Examination of the extra density in this map relative to empty 60S reconstructions revealed the presence of Listerin (orange), a ∼200 Å long sinuous structure snaking from the intersubunit face of 60S to near the ribosome exit tunnel (Figure 3A). The intersubunit surface (Figure 3B) also contained density that we assigned to a P-site tRNA (purple) and NEMF (teal). Confidence in these assignments came from the characteristic shape, position, and size of the tRNA (e.g., Figure 3C) and from NEMF being the only unaccounted 60S-associating component in our purified reaction. This placement of NEMF is consistent with the proposed location of Tae2 in the yeast RQC complex (Lyumkis et al., 2014). Density for segments of the specific nascent polypeptide inside the ribosomal exit tunnel could be visualized and modeled at atomic resolution (Figure 3D), verifying that the reconstruction represented a substrate-occupied 60S complex.

Several insights could be derived from the particle distribution and overall architecture of this data set. First, the majority of 60S particles in the data set contained both Listerin and NEMF. This contrasts with the reaction lacking NEMF, where detection of 60S particles by EM required inclusion of excess eIF6, and only half of these particles contained Listerin (Shao and Hegde, 2014). Thus, consistent with the biochemical analysis, NEMF plays a major role in preventing subunit reassociation. Second, Listerin density in the NEMF-containing map was much better resolved than without NEMF. Indeed, key functional regions near the exit tunnel and intersubunit interface are sufficiently well resolved to permit building of atomic models (Table S1; e.g., Figures S3E and S3F). The improved Listerin density is consistent with its biochemical stabilization by NEMF (Figure 2B). Third, the peptidyl-tRNA was visualized in the NEMF-containing map (Figures 3C and 3D), but not in an earlier map lacking NEMF (Shao and Hegde, 2014). Thus, NEMF appears to stabilize the P-site tRNA via both direct contacts and interactions with the 60S subunit (Figures 3B and 3C), allowing it to effectively take the place of the 40S subunit.

### Structural Analysis of Listerin

**Figure 4:**
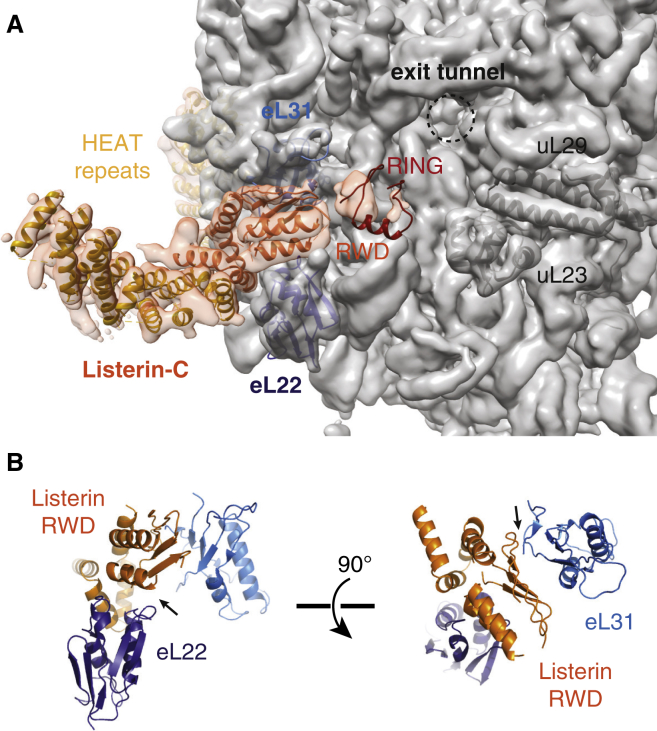
Structural Features of Listerin Bound to the 60S-RNC (A) View of the exit tunnel side of the 60S ribosomal subunit depicting EM density filtered to 5 Å at a threshold that displays secondary structure. The density for Listerin is orange and fit with structural models of HEAT repeats, the RWD domain, and the RING domain. Models for the following ribosomal protein are also fit in position: eL22 (dark blue), eL31 (light blue), uL23 (gray), and uL29 (gray). The exit tunnel is shown as a dashed circle. (B) Atomic model of Listerin’s RWD domain with the neighboring ribosomal proteins eL22 (dark blue) and eL31 (light blue), depicting possible sites of interaction (arrows). See also Figure S4.

The improved resolution of our map permitted the interpretation of several key functional domains with atomic models (Tables 1, S1, and S2). Although high-resolution structural information is not available for any part of Listerin, sequence analysis predicts extensive HEAT repeats (residues 1 to 1,550, using human numbering), an RWD domain (residues 1561 to 1699), and a C-terminal RING domain (residues 1,715 to 1,766). Using a combination of secondary structure prediction and homology searches of structural databases, we generated starting models that were fit and adjusted to the observed Listerin density. The RWD domain could be modeled into a well-resolved region of Listerin density near the ribosome exit tunnel (Figures 4A and S4A–S4C). The position of the RWD domain is unambiguous, with clearly identifiable density for six α helices and four β strands consistent with other RWD domains (Figure S4D), permitting a high-confidence atomic model. Placement of the RWD domain definitively orients Listerin with the C terminus near the exit tunnel and N terminus at the intersubunit interface.

Density corresponding to the C-terminal RING domain, while of insufficient resolution to build an atomic model, can be confidently localized near the RWD domain at the precipice of the exit tunnel (Figure 4A). The relatively weak density for the RING domain implies that it may not interact tightly with the ribosome, consistent with its deletion not affecting ribosome binding (Bengtson and Joazeiro, 2010; Brandman et al., 2012). A dynamic RING, anchored mainly by the adjacent RWD, may provide the requisite flexibility to ubiquitinate diverse clientele at the exit tunnel. From this position, the RING domain can recruit the E2 and position its thioester-linked ubiquitin at an ideal location for attack by primary amines on the nascent polypeptide. The RING domain is ∼90° clockwise around the ribosomal exit tunnel from uL23 and uL29, a common docking site for numerous protein biogenesis factors (Beckmann et al., 2001; Kramer et al., 2002; Pool et al., 2002). This implies that nascent chain ubiquitination may be compatible with stalls that occur during certain processes such as protein translocation into the endoplasmic reticulum.

The RWD domain is the primary point of ribosome contact for Listerin in this region, although direct interactions are limited. The helix formed by residues W1676 to Y1686 closely approaches two loops on the surface of eL22 (Figure 4B, left). On the other side, the most N-terminal β strand of the RWD domain appears to interact with the C-terminal tail of eL31 (Figure 4B, right), where conserved basic residues (K1627 and R1629) on the β strand probably interact with acidic residues at the C terminus of eL31 (Figure S4E).

The direct interaction of the RWD domain with the ribosome probably explains its comparatively high-resolution density in our map. Regions N-terminal to the RWD domain were of sufficient resolution to visualize the characteristic helices of HEAT repeats (Figure 4A). These helices were modeled with a simple poly-alanine backbone, since side-chain information could not be confidently assigned. Regions further N-terminal to this, while clearly HEAT repeats, dropped off in resolution due to presumed flexibility in this area. This entire region of Listerin, which does not contact either the ribosome or other factors, appears to act as a structural spacer, since the overall length is well conserved despite substantial sequence divergence.

**Figure 5:**
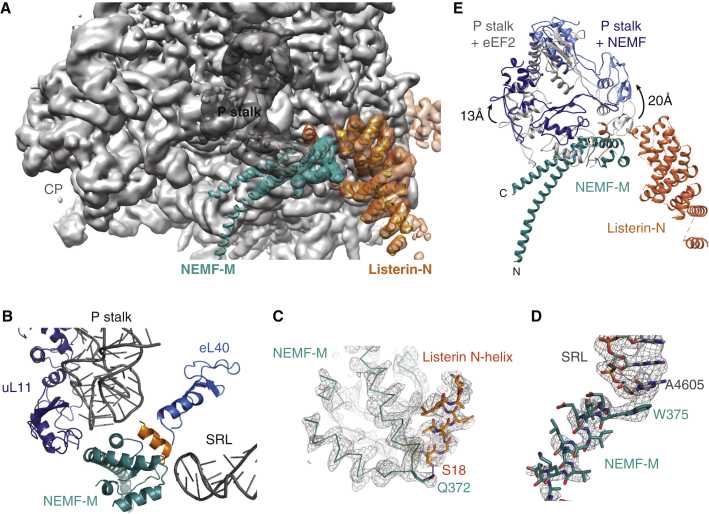
Structural Analysis of NEMF Interactions on the 60S-RNC (A) Side view of the N terminus of Listerin (orange) contacting NEMF (teal) and the 60S subunit (gray) with density for the P stalk in dark gray. De novo models of NEMF’s M-domain and Listerin’s N-terminal helix, along with a poly-alanine model of the helices of Listerin’s N-terminal HEAT repeats is superimposed into the density (filtered at 5 Å). (B) Atomic models of the middle domain of NEMF (NEMF-M, teal), the N-terminal helix of Listerin (orange), eL40 (light blue), uL11 (dark blue), and interacting portions of the 28S rRNA (dark gray). (C) De novo built models of the NEMF-M domain and Listerin's N-terminal helix fit to map density, illustrating a likely interaction. (D) Models fitted to map density illustrating a stacking interaction of W375 of the NEMF-M domain with A4605 of H95/sarcin-ricin loop of the 28S rRNA. (E) View of the M-domain of NEMF (teal) with corresponding positions of the P stalk proteins uL11 (dark blue) and uL10 (light blue) in the map of the 60S subunit in complex with NEMF and Listerin (blue) or of the same proteins in a map of an 80S ribosome bound to eEF2 (gray). See also Figure S5 and Figure S6.

By contrast, the far N-terminal HEAT repeats of Listerin are well-conserved, and our map displayed sufficient resolution to clearly identify helices (Figure 5A). Secondary structure predictions aided the modeling of a poly-alanine backbone throughout this domain, revealing a secondary point of Listerin contact with the ribosome (Figure 5A). The Listerin N-domain appears to make direct contacts with the ribosome and NEMF (see below), which likely stabilize this region of Listerin, as evidenced by its comparatively high resolution in our map.

### NEMF Makes Multivalent Interactions within the RQC

The density corresponding to NEMF can be divided into four prominent domains: the N- and C-lobe connected by a coiled-coil to the middle (M) domain (Figure 3B). By masking only the density for the 60S subunit and NEMF during 3D refinement of our RQC data set, we generated a map that permitted building of an atomic model of the NEMF coiled-coil, its M-domain, and an N-terminal helix of Listerin (Figures 5A and S5A–S5C). This region of NEMF is intimately sandwiched between the P stalk of the ribosome, the sarcin-ricin loop (H95), and two N-terminal helices of Listerin (Figures 5B–5D). These interactions effectively pin Listerin’s N-terminal helix, comprising residues 13–27, to the 60S subunit (Figures 5B and 5C). This configuration explains why 60S-bound NEMF facilities Listerin recruitment: it provides an additional point of contact that cooperates with the RWD-ribosome interaction to tether Listerin at both ends.

NEMF makes three contacts with the ribosome in this area (Figure 5B). The N-terminal helix of the coiled-coil interacts with uL11 before it bends sharply (90°) into the M-domain. The globular M-domain, comprising of three short helices and two β strands (Figures S5A and S5B), contacts 28S rRNA. In particular, W375 of NEMF interacts with the sarcin-ricin loop (Figure 5D). The C-terminal helix of NEMF’s coiled-coil contacts the loop regions of both H43 and H44 of the P stalk, stabilizing it in a defined position. The beginning of this helix is rich in aromatic and basic residues that could form stacking interactions with rRNA bases and electrostatic interactions with the rRNA backbone, respectively (Figure S5B).

The P stalk is ordinarily dynamic and not visualized by cryo-EM unless it is stabilized by a translation-associated GTPase such as eEF2 (Anger et al., 2013; Voorhees et al., 2014). The NEMF interaction with the P stalk, via uL11 and the 28S rRNA, positions it at a site ∼15 Å away from that seen with eEF2 (Figure 5E). The stabilization of the P stalk at this site might contribute to Listerin recruitment by preventing its ability to block the adjacent region occupied by the Listerin N-domain.

Based on the directionality of the coiled-coil helices of NEMF, we could distinguish between and position the N- and C-terminal globular domains of NEMF (Figure 3). The N-lobe of NEMF contains an NFACT-N domain, which has known structural homologs (Burroughs and Aravind, 2014). We therefore created a comparative model using the structure of a fibrinogen binding protein from *Staphylococcus aureus* (PDB ID: 3DOA) and adjusted its fit into the observed density. In this position, the NFACT-N domain appears to contact the anticodon loop of the peptidyl-tRNA via two basic loops (Figure S6A). Sequence analysis has suggested that this domain may have RNA glycosidase activity (Burroughs and Aravind, 2014); whether it in fact does have catalytic activity that participates in NEMF function remains to be determined.

Density for the C-lobe of NEMF is predicted to house two domains of unknown function, NFACT-R and NFACT-C (Burroughs and Aravind, 2014). The resolution of this region is limited, preventing meaningful atomic modeling. Nevertheless, the C-lobe is seen to cup the other side of the tRNA anticodon stem and appears to contact the ribosome at eL5 and 28S rRNA near the central protuberance (Figures 3C and S6B).

This structural analysis not only provides an overall architecture of the mammalian RQC complex, but also identifies the extensive network of interactions among all of the components. NEMF contacts the 60S subunit at multiple distinct sites, most of which are completely inaccessible when the 40S is bound. This, together with two different P-site tRNA interactions, explains how 40S reassociation is efficiently prevented by NEMF (Figure 2D). With the 40S unable to bind, the N-terminal half of Listerin is unobstructed from its eventual position, allowing its recruitment. Listerin’s direct contact with NEMF, along with two different ribosome contacts at either end of the molecule, collectively hold Listerin in an optimal position for nascent polypeptide ubiquitination. Thus, no fewer than eight points of contact between 60S, NEMF, tRNA, and Listerin stabilize the RQC complex.

### Biochemical Analyses of RQC Assembly and Function

**Figure 6:**
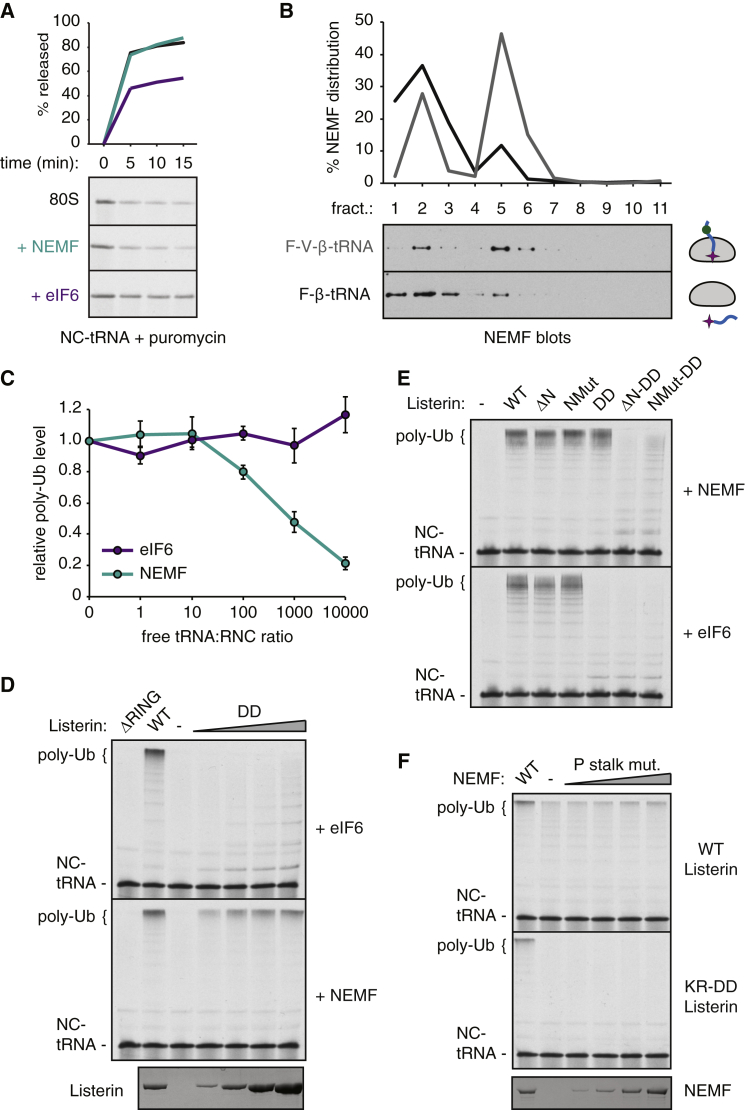
Biochemical Analysis of RQC Assembly and Function (A) Affinity-purified 80S-RNCs were left untreated (black) or incubated with splitting factors in the presence of either NEMF (teal) or eIF6 (purple). Puromycin was then added, the reaction was analyzed by SDS-PAGE at the indicated time points, and the amount of nascent chain released from tRNA by puromycin quantified. (B) Affinity-purified 80S-RNCs were prepared containing a short nascent chain without (F-β tRNA, black) or with (F-V-β tRNA, gray) a small folded domain outside the ribosomal exit tunnel. The RNCs were incubated with 1.2 nM NEMF, 50 nM splitting factors, and energy and analyzed for NEMF recruitment to ribosomes via a 10%–50% sucrose gradient. Upon ribosome splitting, F-β tRNA will “drop out” of the 60S subunit (with ∼50% efficiency), while F-V-β tRNA remains quantitatively trapped. NEMF recruitment to the RNCs is reduced for the drop-off substrate. (C) 80S-RNCs were subjected to ubiquitination reactions with 1.2 nM Listerin, splitting factors, ubiquitination reagents, and energy in the presence of various amounts of free tRNA. One set of reactions contained 1.2 nM NEMF (teal), while the other contained 250 nM eIF6 (purple) to prevent 40S reassociation. Samples were analyzed by SDS-PAGE, autoradiography, and phosphorimaging. The relative amount of poly-ubiquitination compared to the sample without added tRNA was quantified from three independent experiments. Data points represent the mean ± SEM. (D) 80S-RNCs were subjected to ubiquitination reactions with ubiquitination reagents, energy, splitting factors, either 50 nM eIF6 (top) or 1.2 nM NEMF (bottom), and either 1.2 nM wild-type Listerin or increasing amounts of the KR-DD mutant Listerin predicted to abolish the interaction between the RWD domain and eL31 (see Figures 4B and S5D). Reactions with NEMF were for 2 min, while reactions with eIF6 were for 5 min. Autoradiography depicting the nascent chain-tRNA (NC-tRNA) and poly-ubiquitinated species (poly-Ub), and Coomassie staining showing the relative amounts of purified Listerin are shown. (E) Autoradiography of 5 min ubiquitination reactions containing 80S-RNCs, ubiquitination reagents, energy, splitting factors, either 1.2 nM NEMF (top) or 50 nM eIF6 (bottom), and 1.2 nM of different Listerin mutants showing nascent chain-tRNA (NC-tRNA) and poly-ubiquitinated substrate (poly-Ub). (F) Autoradiography of 2 min ubiquitination reactions containing 80S-RNCs, ubiquitination reagents, energy, splitting factors, 1.2 nM of either wild-type (WT) or KR-DD Listerin, and either 1.2 nM wild-type or increasing amounts of a NEMF containing four point mutations in the residues predicted to interact with the P stalk. Nascent chain-tRNA (NC-tRNA) and poly-ubiquitinated substrate (poly-Ub) are indicated. See also Table S3.

With a defined reconstituted system for RQC assembly and ubiquitination, together with structural information for each factor and their interactions, we tested key predictions using functional assays. We first examined the NEMF-tRNA interaction and its importance in sensing occupancy. One implication from the 60S-RQC structure is that NEMF stabilizes the position of P-site tRNA (Figure 3C). This was probed using puromycin, whose reactivity with peptidyl-tRNA occurs at the peptidyl transferase center. 60S-RNCs containing NEMF were reactive with puromycin at levels comparable to intact 80S-RNCs, while 60S-RNCs lacking NEMF were significantly less reactive (Figure 6A). Thus, the peptidyl-tRNA is stabilized from slipping out of the P-site by NEMF, supporting their interaction in the configuration seen in our structure (Figure 3C).

We next tested whether stable NEMF-60S interaction requires the peptidyl-tRNA. This was accomplished by comparing NEMF recruitment under conditions where the peptidyl-tRNA would either remain on the 60S or drop off to a substantial extent during ribosome splitting. We found that NEMF recruitment was substantially reduced for the drop-off substrate (Figure 6B), with the residual binding being explained by incomplete (∼50%) drop-off. Thus, the peptidyl-tRNA plays a key role in recruiting NEMF, and in turn Listerin, selectively to nascent chain-containing 60S subunits.

This predicts that free tRNA should compete for NEMF-dependent Listerin recruitment to reduce nascent chain ubiquitination. Indeed, titration of purified total liver tRNA inhibited NEMF-dependent RNC ubiquitination (Figure 6C) but not NEMF-independent ubiquitination (in which subunit reassociation was artificially prevented with excess eIF6). Importantly, the levels of free tRNA needed to see appreciable (∼50%) inhibition was ∼1,000-fold in excess of RNCs, indicating that this would not be a relevant competitor under physiologic conditions. Thus, while NEMF critically depends on tRNA for occupancy sensing and 60S recruitment, this interaction is tuned to preclude competition by free pools in vivo.

We next turned our attention to Listerin-NEMF-60S interactions that could be visualized at sufficient resolution to permit structure-guided mutagenesis. We first analyzed the Listerin-60S interaction near the exit tunnel. Here, two basic residues in the RWD domain (K1627 and R1629) appear to contact the acidic C-terminal residues of eL31. Mutation of both basic residues to aspartates (Table S3) completely abolished Listerin's ability to ubiquitinate eIF6-stabilized 60S RNCs (Figure 6D, top panel). Approximately 50% activity could be restored by including NEMF in the reaction (Figure 6D, bottom panel), consistent with NEMF interacting with and facilitating Listerin-60S stability (Figures 2B and 3B). To test this, we deleted or mutated the very N-terminal helix of Listerin predicted to contact NEMF. While these mutants had very little effect on their own, they markedly attenuated ubiquitination when combined with the RWD mutation (Figure 6E). Thus, Listerin contains two sites that interact with the 60S ribosomal subunit—one via its C-terminal RWD domain and another via its N terminus that depends on NEMF.

We also tested the importance of NEMF interactions with the P stalk by mutating residues predicted to mediate this interaction and testing its function using either wild-type or RWD mutant Listerin. Ubiquitination was completely abolished when combined with the RWD mutant and inhibited by ∼50% with wild-type Listerin (Figure 6F). The mutant analyses collectively illustrate that NEMF’s interaction with the P stalk facilitates stable Listerin recruitment to this site via its N-terminal helix. This stabilization is minimally sufficient for Listerin-mediated ubiquitination of the nascent chain but is enhanced by the additional RWD interaction near the exit tunnel. Thus, the importance of the P stalk-NEMF-Listerin interaction network is most clearly revealed when the RWD interaction is crippled, while the RWD interaction becomes critical in the absence of NEMF interaction. Even though the individual mutants show subtle effects in these assays, they probably become biologically significant in the context of high deubiquitination activity in vivo (Zhang et al., 2013).

### Working Model and Implications

**Figure 7:**
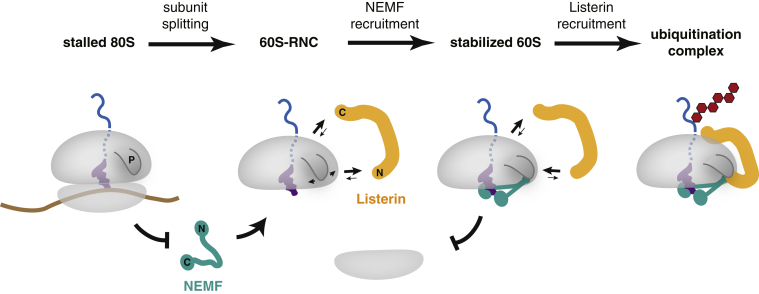
Working Model for Step-Wise Assembly of RQC Ubiquitination Complex Translationally stalled 80S ribosomes, which are inaccessible to both Listerin (orange) and NEMF (teal) binding, are split into subunits by the factors Pelota, Hbs1, and ABCE1 (not displayed). Ribosome splitting exposes the peptidyl tRNA of a trapped nascent chain within the 60S subunit. At this stage, Listerin can potentially bind, but is competed by 40S reassociation and a dynamic P stalk (P). By contrast, NEMF specifically recognizes and binds the peptidyl tRNA-60S interface via its globular N- and C-terminal lobes. Upon binding the 60S-tRNA interface, the coiled coil and M-domain of NEMF bind and stabilize the P stalk in a defined position. This generates an improved binding site for the N terminus of Listerin between NEMF and the 60S, facilitating docking of its C-terminal RWD domain. The ribosome-bound RWD domain positions the ligase domain at the nascent chain exit tunnel, leading to a productive ubiquitination complex.

The cellular, biochemical, and structural experiments described here lead to a mechanistic model for how the Listerin ubiquitin ligase accesses its clients with high fidelity and efficiency during the quality control of stalled translation products (Figure 7). A critical first step is removal of the 40S subunit from 80S-RNCs (Shao and Hegde, 2014; Shao et al., 2013). Our structure reveals the reason for this: essentially all of the contacts made by NEMF and Listerin with the ribosome are obscured on 80S ribosomes. This architecture is similar to that seen in a recent moderate-resolution cryo-EM reconstruction of the yeast RQC complex (Lyumkis et al., 2014). The only accessible site, a contact between Listerin’s RWD domain with eL31 and eL22, is ineffectual for 80S-RNC ubiquitination due to steric clashes of the Listerin N-terminal domain with the 40S subunit. Even with Listerin’s potential flexibility (Lyumkis et al., 2013), this single contact is apparently too transient to permit nascent chain ubiquitination, since isolated 80S-RNCs cannot be ubiquitinated by purified Listerin (Shao and Hegde, 2014). This explains why translating ribosomes are not at risk of promiscuous ubiquitination despite displaying a ligase binding site near the ribosome exit tunnel.

Once the 40S subunit has been removed by ribosome recycling factors, exposure of the intersubunit surface of 60S together with a P-site peptidyl-tRNA efficiently recruits NEMF. This recruitment does not appear to be tightly coordinated with splitting, since a short peptidyl-tRNA is efficiently released from the ribosome during splitting even in the presence of 25-fold excess NEMF (data not shown). This suggests that NEMF binding is a separate event after splitting, since splitting-coupled assembly would lock the tRNA in place and preclude its drop-off. A sequential mechanism ensures that NEMF is loaded only onto those RNCs whose polypeptide has protruded sufficiently from the exit tunnel to access the ligase. Conversely, shorter nascent chains that would be inaccessible for ubiquitination are not trapped, allowing their degradation by other means.

NEMF interaction with 60S-RNCs involves two regions that contact the tRNA and three that contact the ribosome. The tRNA contacts, which appear to be required for RQC complex recruitment in vitro and in cells, explains how 60S occupancy is sensed by NEMF. It is noteworthy that despite three independent contact sites with the 60S, stable NEMF association is nevertheless dependent on P-site tRNA. One attractive explanation may be that the N- and C-lobes of NEMF are normally dynamic in solution, which, together with a dynamic P stalk, would disfavor a productive encounter. The P-site tRNA, by binding to both lobes, may help orient them to permit each lobe’s ribosome interaction. Once these regions of NEMF are bound, the M-domain can then capture the dynamic P stalk and hold it in place.

Stabilization of the P stalk in this location probably minimizes obstruction of the binding site for Listerin’s N-terminal domain. Listerin interaction at this site is stabilized by contacts with both NEMF and the ribosome, which together sandwich Listerin’s N-terminal helices. This sequence of events would explain why NEMF stabilizes Listerin-60S association, while Listerin is not required for NEMF recruitment. The interaction between the RWD domain at the opposite end of Listerin with eL31 and eL22 orients the C terminus such that the RING domain is close to the exit tunnel, optimally positioned for nascent chain ubiquitination. Listerin’s two direct ribosome contacts near its N and C termini, facilitated by its interaction with ribosome-stabilized NEMF, explains its highly stable binding to 60S-RNCs. This is presumably why NEMF enhances processivity of Listerin-mediated ubiquitination.

Thus, the high specificity of Listerin targeting to its clients is encoded at multiple levels. First, the architecture of Listerin itself essentially prevents its ability to access 80S ribosomes. Second, its efficient ribosome interaction requires a second binding site formed by both the ribosome and NEMF, making it strongly dependent on the latter. Even though ubiquitination is clearly possible without NEMF in vitro, the strong competing reactions of subunit reassociation, Listerin dissociation, and possibly deubiquitination would further reduce efficiency in vivo. This may explain why in yeast, the effect of *TAE2* deletion on this pathway is muted relative to *LTN1* deletion, with variable effects on Ltn1 ribosome association (Brandman et al., 2012; Defenouillère et al., 2013). Third, by coupling Listerin recruitment to NEMF, the ligase effectively senses nascent chain occupancy via a proxy whose ribosome interaction depends on the peptidyl-tRNA. In this manner, exposure of the intersubunit interface of an RNC can be communicated to the opposite side of the ribosome to cue ubiquitination.

It is remarkable that all of the interactions within the functional ubiquitination complex, while very stable in sum, are individually extremely weak. For example, Listerin and NEMF have no detectable interaction in solution, tRNA interacts with NEMF so weakly that it is an insignificant competitor, and empty 60S does not seem to prevent NEMF recruitment to bona fide targets. Thus, the avidity gained from an extensive network of interactions stabilizes this complex without interference from any of its constituent parts. This means that quality control in this system is carried out entirely on the basis of context, rather than a single aberrant recognition motif.

This contrasts sharply with quality control of mislocalized secretory pathway proteins, where recognition is based on a single and clearly aberrant parameter: exposure of a long hydrophobic domain intended for burial inside a membrane (Hessa et al., 2011). Quality control during protein folding in the ER or cytosol is more nuanced; the recognition feature(s) of misfolded proteins and the mechanisms that link these features to the ubiquitination machinery are not fully understood despite extensive study. In a process such as ER-associated degradation, numerous weakly interacting factors have been implicated in client selection (Hegde and Ploegh, 2010), but a cohesive picture of their individual roles has yet to emerge. Whether the principle of multiple context-specific weak interactions summing into high-fidelity recognition described here will help explain this and other pathways of quality control remains to be seen.

While our results provide a mechanistic framework into the core ubiquitination steps of the RQC pathway, the events preceding and following these steps remain to be explained. For example, the mechanism by which splitting factors differentiate stalled from translating ribosomes is unclear. Similarly, the exact role of the Cdc48 complex and the mechanism of poly-ubiquitinated nascent chain extraction from 60S is unknown. These are outstanding questions for future studies of this pathway.

## Experimental Procedures

### In Vivo and In Vitro Analyses

Drug treatments of HEK293T cells were with 50 μg/ml CHX, 1 mM puromycin, or DMSO for 30 min followed by extraction of the cytosol with 0.1% digitonin for biochemical analyses. siRNA knockdowns were performed according to standard protocols. In vitro transcription and translation, affinity purifications, recombinant protein production, sucrose gradient analyses, and ubiquitination assays were as before (Shao and Hegde, 2014; Shao et al., 2013). Final concentrations of components in purified reactions were as follows: 5 nM 80S RNC complexes, 1.2 nM Listerin, 1.2–50 nM NEMF as described in individual figure legends, 50 nM splitting factors, 75 nM E1, 250 nM E2, 10 μM ubiquitin, and 1 mM ATP and GTP. Reactions were performed at 32°C for 2–15 min as indicated. See the Supplemental Experimental Procedures for further details.

### Cryo-EM, Image Processing, and Modeling

Affinity purified 80S stalled RNCs were incubated with equimolar amounts of splitting factors, Listerin, NEMF, and 1 mM ATP and GTP for 5 min at 32°C. Ribosomes were isolated via centrifugation and resuspended in a mixture of Listerin and NEMF before being vitrified on EM grids. Automatic data acquisition was conducted on a Titan Krios operated at 300 kV at a magnification of 104,478×. Semiautomated particle picking was conducted with EMAN2 (Tang et al., 2007) before the data sets were processed through RELION (Scheres, 2012; Shao et al., 2013). Homology models were obtained using I-TASSER (Zhang, 2008). Modeling was done with Coot (Emsley et al., 2010) and refined with REFMAC optimized for EM maps (Amunts et al., 2014). Details can be found in the Supplemental Experimental Procedures. Chimera (Pettersen et al., 2004) and PyMOL (http://www.pymol.org) were used to visualize maps and models and to generate figures.

## Author Contributions

S.S. performed biochemical experiments and collected and analyzed cryo-EM data; A.B. did all structural modeling; B.S. contributed sequence analyses; R.S.H. oversaw the project. All authors interpreted results; R.S.H. and S.S. wrote the paper with input from all authors.

## Figures and Tables

**Table 1:** Refinement and Model Statistics

Data Collection	
Particles	63,826
Pixel size (Å)	1.34
Defocus range (μm)	1.5–3.5
Voltage (kV)	300
Electron dose (e-Å^−2^)	35

**Model Composition**	

Nonhydrogen atoms	138,980
Protein residues	6,725
RNA bases	3,938
Ligands (Zn^2+^/Mg^2+^)	5/159

**Refinement**	

Resolution (Å)	3.60
Map sharpening B factor (Å^2^)	−99.3
Average B factor (Å^2^)	65.5
FSC_average_	0.88

**Rms deviations (RMSD)**	

Bonds (Å)	0.008
Angles (°)	1.44

**Validation (proteins)**	

Molprobity score	3.09 (85^th^ percentile)
Clashscore, all atoms	10.6 (97^th^ percentile)
Good rotamers (%)	83.1

**Ramachandran plot**	

Favored (%)	87.2
Outliers (%)	2.6

**Validation (RNA)**	

Correct sugar puckers (%)	94.7
Good backbone conformations (%)	63.2

Chains that were placed in the density by rigid body fitting were not included during final refinement and are annotated as “docked” in the deposited coordinate file. See also Tables S1 and S2.

## Accession Numbers

The cryo-EM map reported here can be found under the EMDB accession number 2832. Combined atomic coordinates of corresponding structural models have been deposited with the PDB accession number 3J92.

## Supplemental Information

Document S1. Figures S1–S6, Tables S1–S3, and Supplemental Experimental Procedures

Document S2. Article plus Supplemental Information
