# Supplementary material for: Structure and Assembly Pathway of the Ribosome Quality Control Complex
Source: Mol Cell. 2015 Feb 5;57(3):433–44. doi: 10.1016/j.molcel.2014.12.015 (PMC4321881; doi:10.1016/j.molcel.2014.12.015)
Supplement: Document S1. Figures S1–S6, Tables S1–S3, and Supplemental Experimental Procedures [file mmc1.pdf]

Molecular Cell, Volume *57*

Supplemental Information

Structure and Assembly Pathway

# of the Ribosome Quality Control Complex

Sichen Shao, Alan Brown, Balaji Santhanam, and Ramanujan S. Hegde

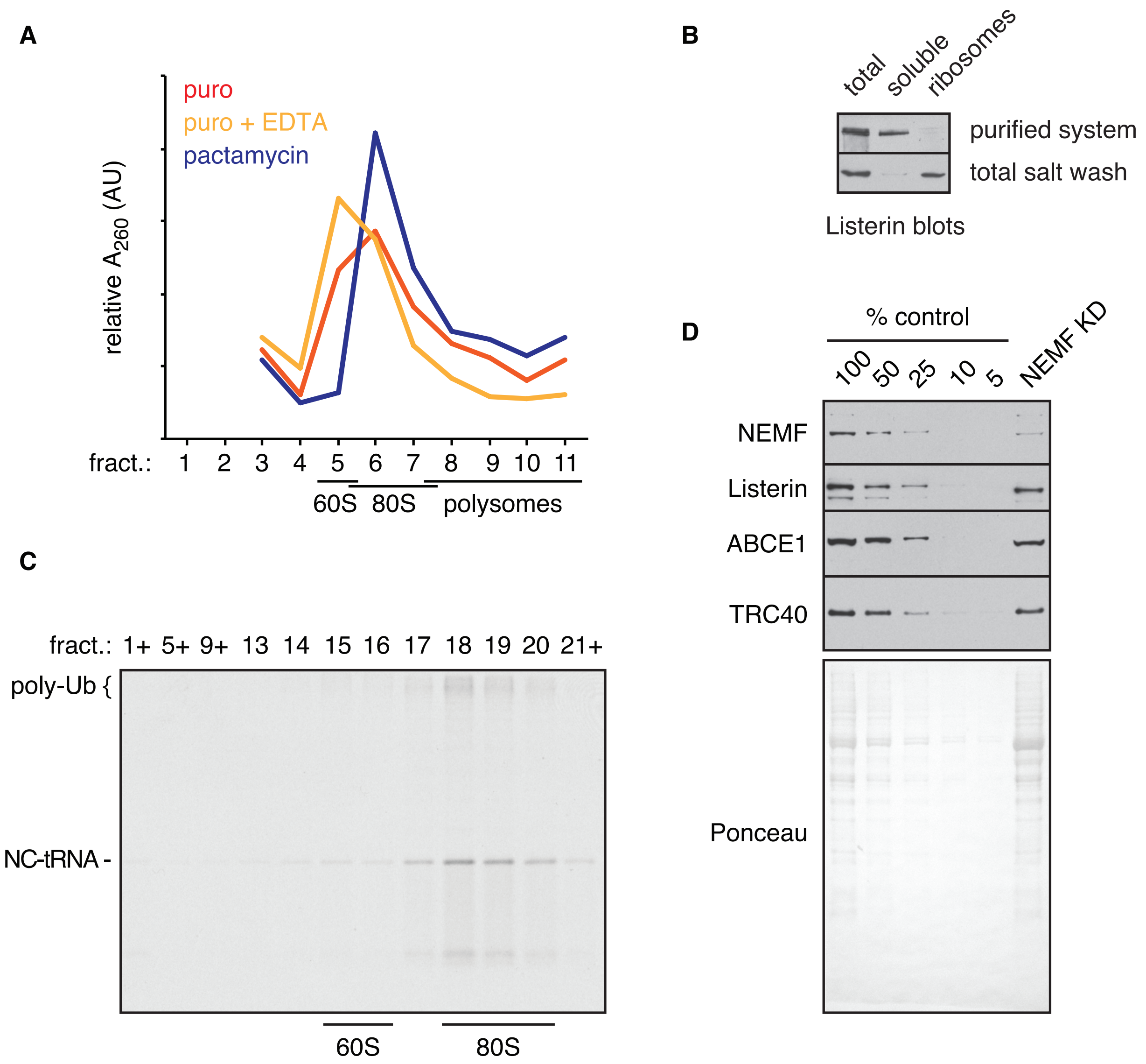

Fig. S1. Additional analysis of Listerin and NEMF (related to Fig. 1)
(A) HEK293T cells were treated with 1 mM puromycin or 0.2 µM pactamycin for 15 min at 37°C and then lysed as in Fig. 1a-1c. After lysis, the puromycin-treated lysate was divided and one half was adjusted to 17.5 mM EDTA (2.5 mM in excess of the MgAc2 present). All lysates were then size fractionated on a 10-50% sucrose gradient. A260 measurements were taken and plotted to assay for migration of ribosomes throughout the gradient. Both puromycin and pactamycin cause a distinct shift in the A260 profile from polysomes to smaller fractions (compare to Fig. 1b). Puromycin inhibits translation elongation by releasing nascent chains, allowing for ribosome splitting to generate 60S and 40S subunits. Pactamycin inhibits translation initiation, trapping initiation complexes on 80S monosomes. Thus, the sharp peak in fraction 6 with pactamycin is a marker for 80S. Relative to this standard, the puromycin sample has a clear shoulder in fraction 5, where 60S would migrate. This was verified by a greater shift to fraction 5 upon EDTA treatment, which dissociates ribosomal subunits. **(B)** 5 nM of affinity purified stalled radiolabeled 80S ribosome-nascent chains (RNCs) were incubated for 10 min at 32°C with either total high salt wash isolated from native rabbit reticulocyte ribosomes, or purified factors (50 nM Hbs1, 50 nM Pelota, 50 nM ABCE1, and 1.2 nM Listerin). Both reactions contained 75 nM E1, 250 nM UbcH5a, 10 µM ubiquitin, 1 mM ATP, 1 mM GTP, 12 mM creatine phosphate, and 20 µg/mL creatine kinase. The reactions were centrifuged to sediment ribosomal particles, and equal amounts of the total reaction (T), supernatant (S) and ribosomal pellet (P) were analyzed by SDS-PAGE and immunoblotting for Listerin. Note that while both reactions can mediate stalled RNC ubiquitination (Shao and Hegde, 2014), only the total ribosome salt wash results in efficient Listerin recruitment to the ribosomal pellet. This supports the hypothesis that the salt wash contains a factor not present in the purified system that stabilizes Listerin on ribosomes. **(C)** Affinity purified stalled radiolabeled 80S ribosome-nascent chains (RNCs) were incubated for 10 min at 32°C with purified splitting factors (50 nM Hbs1, 50 nM Pelota, 50 nM ABCE1), ubiquitination reagents (75 nM E1, 250 nM UbcH5a, 10 µM ubiquitin), energy (1 mM ATP, 1 mM GTP, 12 mM creatine phosphate, and 20 µg/mL creatine kinase) and 1.2 nM Listerin. The reaction was separated on a 10%-30% sucrose gradient and the resulting fractions analyzed by SDS-PAGE and autoradiography. The unmodified tRNA-linked nascent chain (NC-tRNA) and poly-ubiquitinated products (poly-Ub) are indicated. Fractions corresponding to 60S and 80S are indicated. Although Listerin-mediated ubiquitination in a purified system requires 40S removal (Shao and Hegde, 2014), the resulting ubiquitinated products end up migrating in 80S fractions. This suggests that 40S re-association in the purified system is a strong competing event, and that Listerin by itself is unable to prevent this even though it can associate with 60S subunits sufficiently long to mediate ubiquitination. **(D)** HEK293T cells were treated with 10 nM control or NEMF siRNA for 30 h. Serial dilutions of cytosol from control cells were compared to cytosol from NEMF knockdown cells by immunoblotting for the indicated proteins. NEMF was reduced to ~25% of control. Ponceau staining displays total protein content in the extracts.

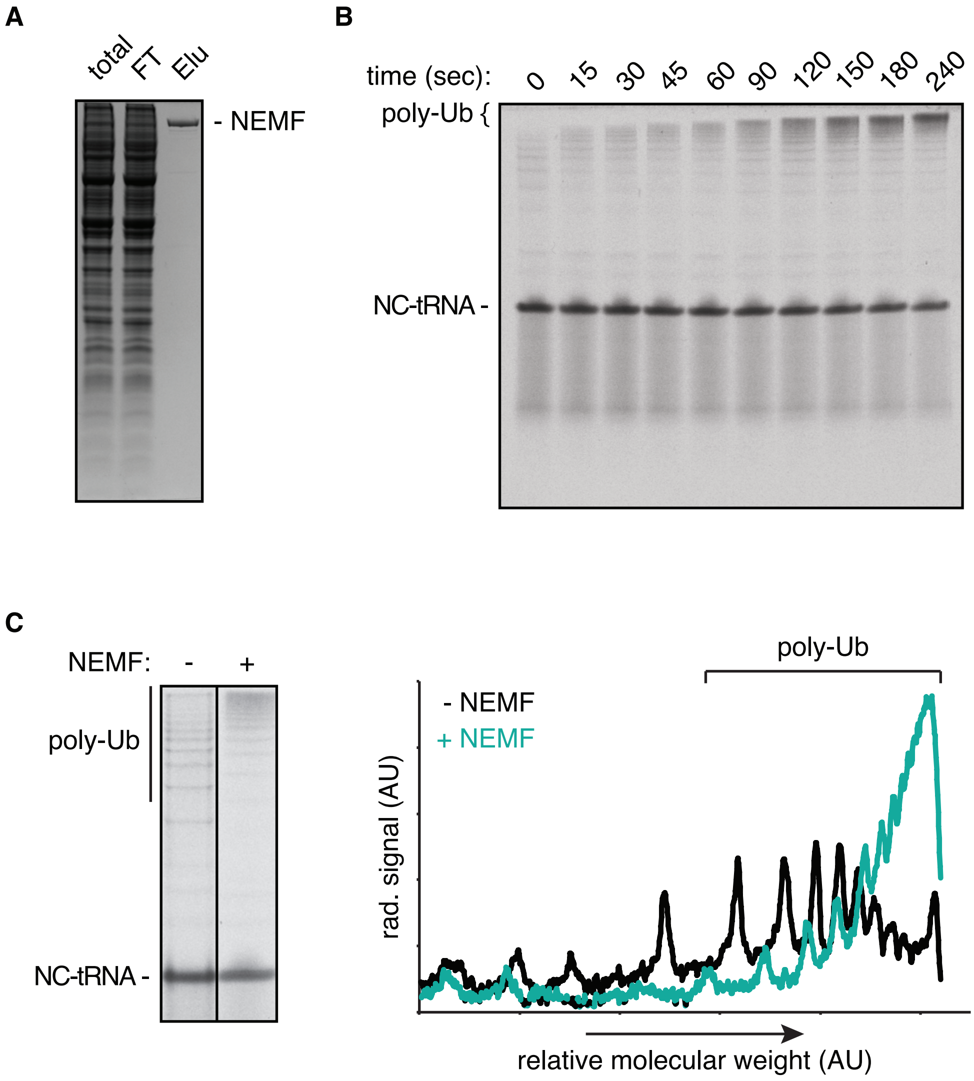

Fig. S2. Effect of NEMF on ubiquitination (related to Fig. 2)
(A) Coomassie stain of fractions from the purification of Flag-tagged human NEMF from transiently transfected HEK293T cell lysates. FT is flow-through, and Elu is eluate. **(B)** Reactions containing radiolabeled 80S-RNCs, 1.2 nM Listerin, 1.2 nM NEMF, 50 nM splitting factors, 75 nM E1, 250 nM E2, 10 µM ubiquitin, and energy were assembled on ice and incubated for the indicated times at 32°C. The unmodified tRNA-linked nascent chain (NC-tRNA) and poly-ubiquitinated products (poly-Ub) are indicated, revealing that ubiquitination in the purified system containing both Listerin and NEMF is very fast and processive. Note that ubiquitination is detectable even at the zero time point where the aliquot was taken directly from ice without further incubation. **(C)** In vitro ubiquitination reactions as in panel B were incubated for 2 min at 32°C without or with 1.2 nM NEMF. Shown is the autoradiograph of the products (left) and densitometry of the entire lane above the non-modified NC-tRNA substrate. Peaks in the graph moving towards the right correspond to progressively longer ubiquitin chains. Note that the reaction containing NEMF (teal) results in much more processive ubiquitination, as evidenced by the marked shift toward longer ubiquitin chains.

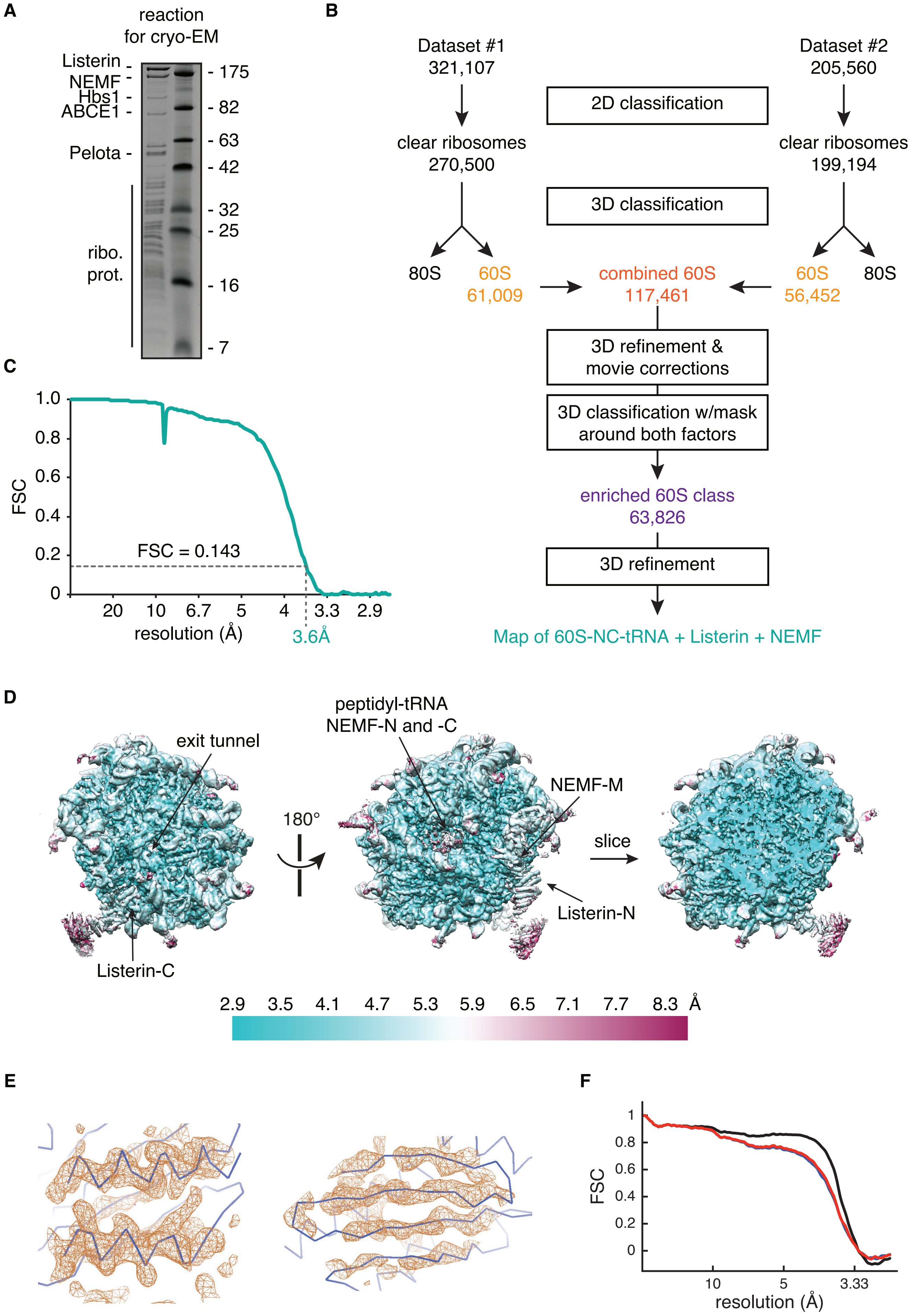
A
reaction
for cryo-EM
Listerin
NEMF
Hbs1
ABCE1
Pelota
ribo.
prot.
175
82
63
42
32
25
16
7
B
Dataset #1
321,107
Dataset #2
205,560
2D classification
clear ribosomes
270,500
clear ribosomes
199,194
3D classification
80S
60S
61,009
combined 60S
117,461
60S
56,452
80S
3D refinement &
movie corrections
3D classification w/mask
around both factors
enriched 60S class
63,826
3D refinement
Map of 60S-NC-tRNA + Listerin + NEMF
C
1.0
0.8
0.6
0.4
0.2
0
FSC
FSC = 0.143
20
10
6.7
5
4
3.3
2.9
resolution (Å)
3.6Å
D
exit tunnel
peptidyl-tRNA
NEMF-N and -C
NEMF-M
180°
slice
Listerin-N
Listerin-C
2.9
3.5
4.1
4.7
5.3
5.9
6.5
7.1
7.7
8.3
Å
E
F
1
0.8
0.6
0.4
0.2
0
FSC
10
5
3.33
resolution (Å)

Fig. S3. 60S-RNC cryo-EM specimen preparation and analysis (related to Fig. 3)

(A) Coomassie stained gel of the reaction analyzed by cryo-EM containing stalled RNCs, splitting factors, energy, Listerin and NEMF. The individual proteins are indicated. Note that while splitting factors are included in reaction to generate the RQC complex, they act only on 80S ribosomes and are therefore not visualized in the final map of the 60S-nascent chain-NEMF-Listerin complex. **(B)** Flowchart of the data analysis scheme to generate the final EM map of the 60S-RNC in complex with Listerin and NEMF. The number of ribosomal particles after each step that were carried forward in the workflow is indicated. Two datasets, collected on separate days as described in the Extended Experimental Procedures were subjected to initial 2D and 3D classification separately to isolate 60S ribosomal particles. These were combined, refined and reclassified to enrich for populations containing Listerin and NEMF. The refinement of these 60S ribosome particles enriched for Listerin and NEMF occupancy generated the final map presented. **(C)** FSC curve of the EM map, demonstrating an overall resolution of 3.6 Å according to gold-standard FSC criteria. **(D)** Local resolution of the 60S-RNC map. Locations of NEMF, peptidyl tRNA, Listerin and the ribosomal exit tunnel are indicated. Note that the core of the ribosome is very well-resolved, while the associated factors are at lower resolution. **(E)** Cryo-EM density (orange mesh) corresponding to Listerin's RWD domain superimposed on the backbone of the resulting atomic model, demonstrating clear visualization of secondary structural features, including the register of α-helices and the separation of β-strands. **(F)** Cross-validation was used to monitor overfitting. Fourier shell correlation (FSC) curves were calculated between the refined model and the final map (black), and with the self (blue) and cross-validated (red) correlations.

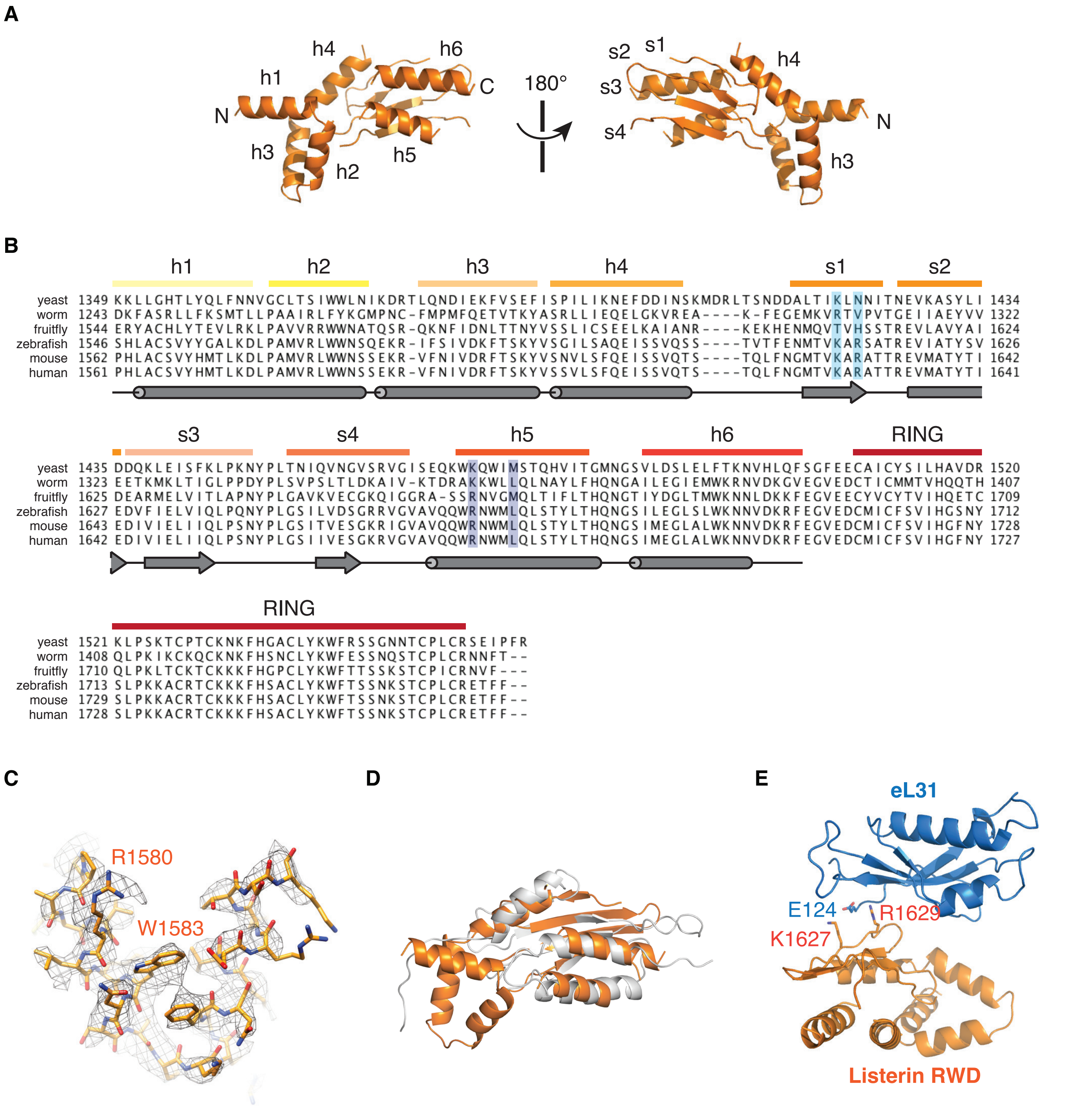

Fig. S4. Atomic model of Listerin RWD domain (related to Fig. 4)
(A) Atomic model of the RWD domain of Listerin with α-helices (h1-6) and β-strands (s1-s4) numbered from the N- to C-terminus. **(B)** Sequence alignment of the C-terminal region of Listerin from yeast, C. elegans, fruitfly, zebrafish, mouse, and human. Secondary structure elements from the atomic model of the RWD domain from panel C and the C-terminal RING domain are labeled (top). Secondary structure prediction of the sequence (psipred) is displayed for comparison (below). Residues likely to interact with eL31 (light blue) and eL22 (dark blue), as visualized in Fig. 4b, are highlighted. **(C)** Demonstration of the fit of the de novo model of Listerin's RWD domain to map density, illustrating the assignment of bulky residues. **(D)** Overlay of the de novo model of Listerin's RWD domain with the solution structure of the RWD domain from RNF25 (PDB ID: 2DAY), predicted by PDBeFold to be the most structurally similar model. **(E)** Representation of potential interaction between K1627 and R1629 of Listerin's RWD domain with the C-terminus of eL31 (E124).

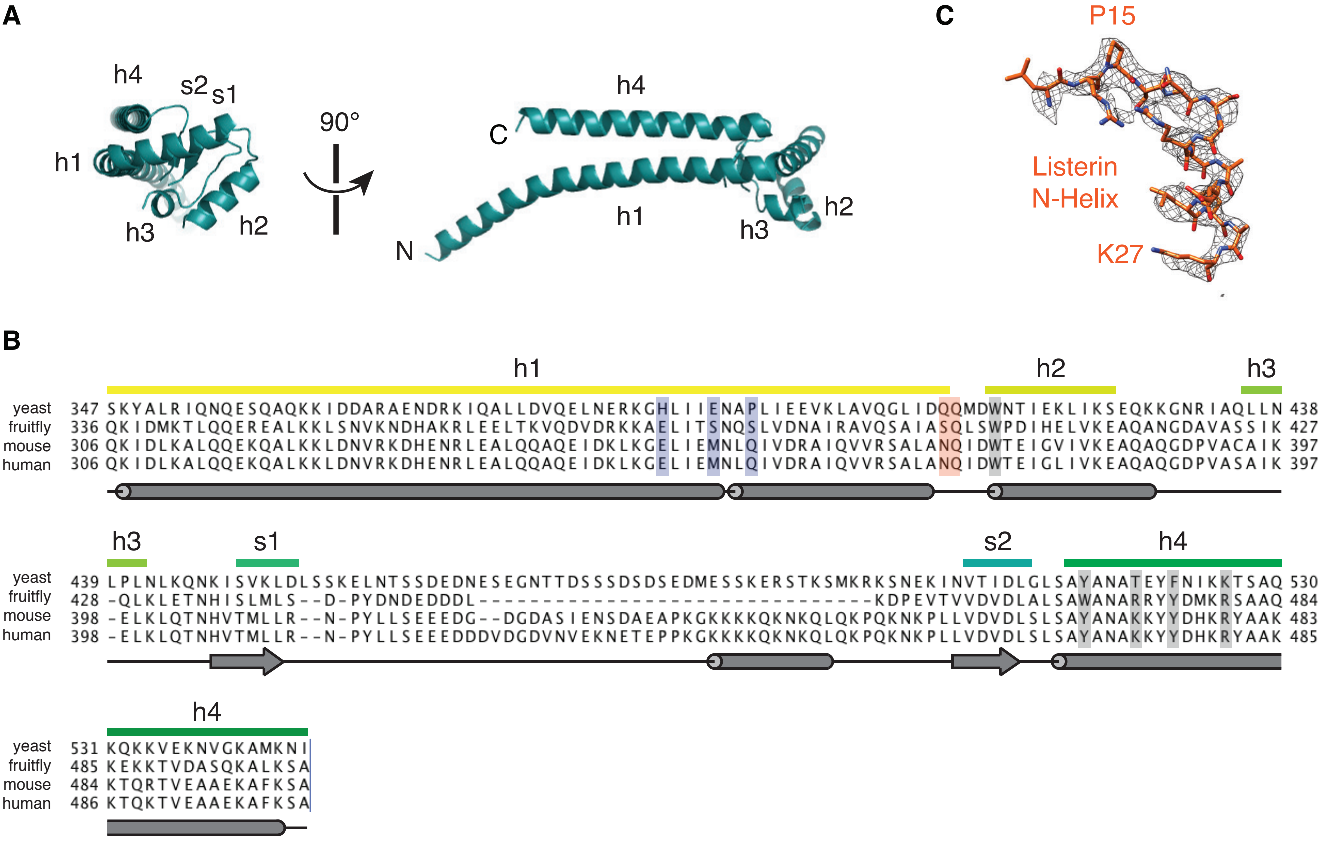

Fig. S5. Atomic model of the NEMF-M domain (related to Fig. 5)
(A) Atomic model of the coiled-coil and M domain of NEMF with α-helices (h1-h4) and β-strands (s1-s2) numbered from N- to C-terminus. Helices 1 and 4 form the long coiled coil region that connects the globular M-domain with the N- and C-lobes of NEMF. **(B)** Sequence alignment of the coiled-coil and M-domain region of NEMF from yeast, fruitfly, mouse, and human. Secondary structure elements from the atomic model in panel A are labeled for reference. Secondary structure prediction of the sequence (psipred) is displayed for comparison (below). Residues that are likely to make interactions with uL11 (dark blue) and rRNA of the large ribosomal subunits (gray) as depicted in Fig. 5b are highlighted. **(C)** Fit of the de novo model of Listerin's N-terminal helix into map density, illustrating assignment of individual residues.

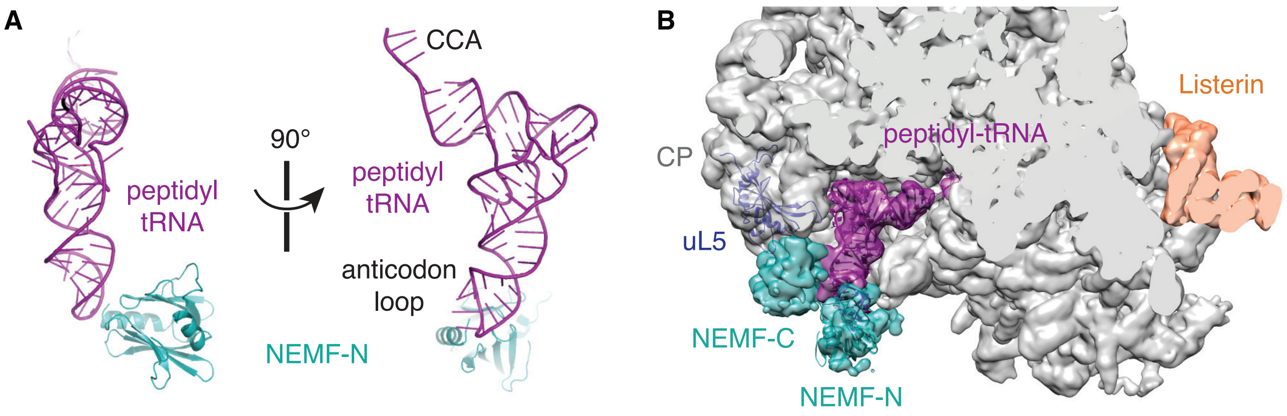

Fig. S6. Interactions of NEMF N- and C-terminal lobes (related to Fig. 5)
(A) Models for the relative positions of the peptidyl-tRNA (purple) and the NFACT-N domain in the N-terminal globular lobe of NEMF (NEMF-N, teal). **(B)** Cut-away view of the 60S-NEMF-Listerin map depicting peptidyl-tRNA density (purple) and density for the globular N- and C-terminal domains of NEMF (teal). Atomic models for the tRNA and the NFACT-N domain of NEMF are superimposed into the density, demonstrating how the NFACT-N domain is likely to directly contact the anticodon stem and loop of the P-site tRNA. On the other side of the tRNA, the density for the C-terminal lobe of NEMF is poorly resolved, but also clearly contacts the tRNA stem and the central protuberance (CP) of the ribosome, potentially making specific contacts with rRNA and uL5 (blue).

Supplemental Tables

Table S1. Factors included in the final model. All other chains are from the mammalian ribosome (PDB ID: 4W1Z and 4W20) (related to Table 1)

Factor	Uniprot ID	Chain ID	Built Residues	Source of model
uL10	P05388	s	5-202	PDB ID: 3J3B
uL11	P30050	t	1-163	PDB ID: 3J3B
NEMF N domain	O60524	u	39-168	I-TASSER comparative model
NEMF M domain	O60524	v	306-386; 394-411; 461-501	Built *de novo*
Listerin N-terminal helix	O94822	w	13-27	Built *de novo*
Listerin N-terminal HEAT repeats	O94822	x	Unassigned	Modeled as idealized helices
Listerin C-terminal HEAT repeats	O94822	y	Unassigned	Modeled as idealized helices
Listerin RWD	O94822	z	1561-1615; 1623-1656; 1659-1670; 1676-1688; 1694-1709	Built *de novo*
Listerin RING	O94822	0	1730-1765	I-TASSER comparative model
Stalled peptide	-	1	10-24	Built *de novo*
tRNA	-	2	1-76	PDB ID: 2J00

Table S2. Refinement statistics for the M domain from NEMF and the RWD domain from Listerin that were modeled *de novo* into the map density. To validate the fit, the registry was shifted by one residue in both directions and the statistics recalculated (related to Table 1)

	**NEMF M domain**			**Listerin RWD**		
Registry	**-1**	**0**	**+1**	**-1**	**0**	**+1**
$FSC_{average}$	0.73	0.77	0.72	0.66	0.77	0.68
RMSD bonds (Å)	0.017	0.012	0.019	0.016	0.012	0.016
RMSD angles (°)	2.12	1.76	2.22	2.28	1.80	2.27

Table S3. Listerin and NEMF mutants (related to Fig. 6)

**Name**	**Mutation**
Listerin DD	K1627D, R1629D
Listerin DN	deletion of residues 13-27
Listerin NMut	P15A, N17A, S18A
NEMF P stalk Mut	Y470A, K474A, Y477A, R481A

Extended Experimental Procedures

Plasmids, siRNAs, and antibodies

SP64-based constructs encoding non-tagged and epitope tagged versions of VHP-β and Sec61β, mammalian expression constructs for Hbs1, Hbs1-DN, ABCE1, and Listerin, and bacterial expression constructs for eIF6 and Pelota have been described (Shao and Hegde, 2014; Shao et al., 2013). The open reading frames of TCF25 and NEMF were cloned into a pcDNA3.1 mammalian expression vector containing an N-terminal 3X Flag tag using standard methods. Listerin and NEMF mutants were generated using Phusion mutagenesis according to established protocols. Silencer Select control and NEMF siRNAs were obtained from Life Technologies. Anti-Listerin (Abcam), anti-L9 and anti-S16 (Santa Cruz) have been described (Shao et al., 2013; Shao and Hegde, 2014). TRC40 antibody was generated as described (Stefanovic and Hegde, 2007); antibodies against Hbs1 and ABCE1 have been described; rabbit polyclonal antibody against NEMF was generated with a KLH-conjugated peptide antigen (PGKVKVSAPNLLNVKRK, Cambridge Research Biomedicals). Hbs1, ABCE1, and NEMF antibodies were further affinity purified using the corresponding peptide epitope obtained from Genscript according to standard protocols. Anti-Flag resin and 3X Flag peptide were from Sigma.

Tissue culture analyses

Drug treatments (mock treatment with DMSO, 50 μg/ml cycloheximide, 1 mM puromycin, or 0.2 μM pactamycin) were for 15 minutes at 37°C on actively growing (~50% confluent) HEK293T cells. Cells were washed once with PBS, the cytosol extracted in 25 mM Hepes pH 7.4, 125 mM KAc, 15 mM $MgAc_2$, 100 μg/mL digitonin, 50 μg/mL cycloheximide , 40U/mL RNasin (Promega), 1 mM DTT, 1X EDTA-free protease inhibitor cocktail (Roche), clarified, and subjected to sucrose gradient fractionation as previously described (Shao et al., 2013). siRNA treatments with 10 nM siRNA was performed with Lipofectamine RNAiMax (Life Technologies) according to vendor-recommended procedures for 30 hours at 37°C before drug treatments, lysis, and processing as described above.

Purification of recombinant proteins

Purification of eIF6, Pelota, Listerin, ABCE1, and Hbs1 have been described (Shao and Hegde, 2014; Shao et al., 2013). Listerin mutants were purified exactly the same as wildtype Listerin. 3X Flag-tagged NEMF (and mutants) and TCF25 were transfected into HEK293T cells, which were passaged once and cultured for 3 days before harvest. Cells were lysed in 50 mM Hepes pH 7.4, 150 mM KAc, 4 mM $MgAc_2$, 1% Triton X-100, 1 mM DTT, and 1X protease inhibitor cocktail (Roche). The clarified supernatant was incubated with anti-Flag resin at 4°C for 1 hour before being washed sequentially in lysis buffer, lysis buffer containing 400 mM KAc, and elution buffer (50 mM Hepes pH 7.4, 100 mM KAc, 5 mM $MgAc_2$, 1 mM DTT). Two sequential elutions were carried out with 100 μg/mL 3X Flag peptide in elution buffer at room temperature for 30 min each.

In vitro transcription, translation, and affinity purifications

Transcript preparation of stalled and drop-off substrates were as before (Shao and Hegde, 2014; Shao et al., 2013). In vitro translation reactions in RRL were performed as previously described. Translation reactions were typically for 15-20 min at 32°C supplemented with either ^{35}S-methionine or 40 μM cold methionine. To generate stalled 80S-nascent chains, ~50 nM of dominant negative Hbs1 was added 7 minutes into the translation reaction to prevent splitting. Affinity purification of salt-washed 80S-nascent chain complexes were as before (Shao and Hegde, 2014). For most assays and reactions for cryo-EM analysis, eluted RNCs

were concentrated by centrifugation and resuspended in 1/10th the original elution volume, yielding a final concentration of ~100 nM.

Ubiquitination assays and sucrose gradient analyses

Unless otherwise indicated, ubiquitination reactions were with 5 nM affinity purified 80S ribosome-nascent chain complexes, 1.2 nM each of Listerin and NEMF; 50 nM each of Hbs1, ABCE1, and Pelota; 75 nM E1, 250 nM E2 (UbcH5a), 10 μM tagged ubiquitin (Boston Biochem), and 1X energy regenerating system (1 mM ATP, 1 mM GTP, 12 mM creatine phosphate, 20 μg/mL creatine kinase) in 50 mM Hepes pH 7.4, 100 mM KAc, 5 mM $MgAc_2$, 1 mM DTT. Total tRNA isolated from pig liver as described (Sharma et al., 2010) was titrated into reactions at concentrations ranging from 120 ng/mL to 1.2 mg/mL. Centrifugation to isolate ribosomes was at 70,000 rpm for 30 min in a TLA 120.1 rotor. Sucrose gradient analysis for total ribosome association was either with 200 μl reactions layered onto a 2 mL 10-50% sucrose gradient spun for 1 hr at 55,000 rpm in a TLS-55 rotor or with 20 μl reactions on a 200 μl 10-50% sucrose gradient spun for 30 min at 55,000 rpm in a TLS-55 rotor (Beckman Coulter) to yield eleven fractions. High-resolution sucrose gradients were performed with 200 μl reactions on 4.8 mL 10-30% sucrose gradients spun for 2 hr at 50,000 rpm in a MLS-50 rotor to yield 25 fractions. For samples that required additional concentration, individual fractions were subjected to TCA precipitation according to established protocols (Shao et al., 2013) prior to SDS-PAGE and immunoblotting analyses.

Electron cryo-microscopy and image processing

Samples for cryo-EM were prepared by translating a truncated mRNA encoding a 3X tandem Flag tag followed by the open reading frame of Sec61β containing the autonomously folding domain of villin headpiece (VHP) as previously described (Shao and Hegde, 2014; Shao et al., 2013). An excess of dominant negative Hbs1 was added 7 min after the start of the translation reaction to prevent ribosome splitting and the reaction allowed to proceed for an additional 18 minutes. Reactions were adjusted to 750 mM KAc and spun through a high salt sucrose cushion for 1 hr at 100,000 rpm in a TLA100.3 rotor (Beckman Coulter). Ribosomal pellets were resuspended in 50 mM Hepes, pH 7.4, 100 mM KAc, 5 mM $MgAc_2$, 1 mM DTT and subjected to affinity purification with anti-Flag resin as previously described. After washing and elution with Flag peptide, the eluted ribosome-nascent chains (RNCs) were directly incubated with equimolar amounts of Hbs1, Pelota, ABCE1, Listerin, NEMF, in the presence of 1 mM ATP and GTP for 5 min at 32°C before being centrifuged for 30 min at 75,000 rpm in a TLA120.2 rotor to re-isolate ribosomes. The ribosomal pellet was resuspended in buffer supplemented with Listerin and NEMF, adjusted to 120 nM, and directly frozen onto glow-discharged R2/2 EM grids (Quantifoil) coated with a continuous layer of carbon. Samples containing TCF25 were prepared identically except for the inclusion of TCF25 at levels equimolar to Listerin and NEMF in all incubation steps.

Automated data collection (EPU software, FEI) was conducted on a Titan Krios operated at 300 kV at 104,478X magnification. One second exposures yielding a total dose of 35 electrons/Å^2 were collected with defocus values ranging from 1.5 to 3.5 μm. Semi-automated particle picking was performed with EMAN2 (Tang et al., 2007). All datasets were subsequently processed through RELION (Scheres, 2012). Initial datasets as described in Fig. S3b were subjected to 2D classification to pick clear ribosomal particles. These particles were then subjected to 3D classification. Classes containing clear 60S ribosomes were then independently refined and corrected for movement and radiation damage with movie processing (Bai et al., 2013). Individual datasets containing polished particles after movie correction were then subjected to additional 3D classification without or with masks around

either Listerin density only or around both Listerin and NEMF density to enrich for occupancy of the factors. Enriched classes resulting from this round of classification were then refined again to produce initial maps. Initial modeling was done on maps generated from the dataset resulting from the reaction containing only Listerin and NEMF. Secondary structure for the N- and C-terminal HEAT repeats of Listerin was assigned and an initial model of the RWD domain was built using the map produced from the enriched class after classification using a mask around Listerin only. Secondary structure of the NEMF M-domain was assigned from the map generated from the particles enriched by classification after masking both Listerin and NEMF densities.

To improve resolution, 60S particles identified after initial 3D classification of two datasets were combined, yielding a total of 117,461 particles for 3D refinement and movie correction. The resulting polished particles were subjected to another round of 3D classification with a mask around both NEMF and Listerin density to enrich for occupancy. The resulting enriched class containing 63,826 particles was refined to produce the final map displayed in all figures and used for modeling. Atomic models of the NEMF-M domain was improved using a map that was refined using the same particles with a mask around the 60S and the NEMF density only.

Structural modeling
Initially, the model of the 60S ribosomal subunit from *Sus scorfa* (PDB ID: 4W1Z and 4W20) was placed into the density map of rabbit 60S using Chimera (Pettersen et al., 2004). The ribosomal protein eL41, which spans the interface between the large and small subunits, is absent from our reconstructions and was deleted from the model. Due to the binding of NEMF and Listerin, the P stalk occupies a different position from that in the porcine model and was fit as a rigid body into the density using Coot (Emsley et al., 2010). Density corresponding to the P stalk proteins uL10 and uL11 was also present in our reconstruction and were interpreted with models from the human ribosome (PDB ID: 3J3B) (Anger et al., 2013). The tRNA bound at the P site was modeled using PDB ID: 2J00. Density for the stalled peptide within the exit tunnel was well-resolved and was built manually using Coot.

To avoid over-interpretation of the density, we have utilized three different types of atomic models that are selected to reflect the resolution apparent in that region. For well-resolved regions of density where side chain information is present we have built all-atom models; for less well-resolved density, but where homologous structures have been solved to high-resolution, we have generated comparative models and docked these into the density, and for regions where no high-resolution structural information is present we have modeled idealized fragments of secondary structure with poly-alanine backbones. All comparative models were generated using I-TASSER (Zhang, 2008).

The density for Listerin is highly heterogeneous, with the N- and C-termini that contact the ribosome best resolved. A full-atom model of the N-terminal helix was built, with the remaining N-terminal HEAT repeats modeled as idealized helices. The central HEAT repeats were not modeled, but the C-terminal HEAT repeats were again modeled with idealized helices. To help identify helices, we low-pass filtered the map to 5Å. Density for the RWD domain is well resolved and an all-atom model was built using a comparative model as an initial template. Registry was assigned using side chain information and predictions of secondary structure. The RING domain was modeled by docking a comparative model.

A comparative model of the NEMF N-terminal domain was generated and fit to the density in Coot. The NEMF-M domain was built *de novo* into the density using secondary structure predictions to help guide model building. The positions of bulky side chains were used both for determination and validation of the correct assignment. The C-terminal domain of NEMF could not be interpreted with a model.

Refinement
Restrained all-atom refinement for the 60S subunit bound to the NEMF-M domain and Listerin RWD domain was performed in REFMAC v5.8 optimized for fitting to EM density maps (Brown et al., 2015) Models that were placed into lower resolution regions of the reconstruction were subjected to rigid body refinement only (Table S1). Secondary structure restraints were generated with ProSMART (Brown et al., 2015) and base pairing and plane parallelization restraints with LIBG (Brown et al., 2015). The fit-to-density was monitored through the $FSC_{average}$, and the final model was validated using MolProbity (Chen et al., 2010). The absence of over-fitting was confirmed using cross-validation (Brown et al., 2015). Model building and refinement statistics are given in tables S1-S3.

Supplemental References

Brown, A., Long, F., Nicholls, R.A., Toots, J., Emsley, P. & Murshudov, G. (2015). Tools for macromolecular model building and refinement into electron cryo-microscopy reconstructions. Acta Cryst. D71, doi:10.1107/S1399004714021683

Chen, V.B., Arendall, W.B., Headd, J.J., Keedy, D.A., Immormino, R.M., Kapral, G.J., Murray, L.W., Richardson, J.S., and Richardson, D.C. (2010). MolProbity: all-atom structure validation for macromolecular crystallography. Acta Crystallogr. D Biol. Crystallogr. *66*, 12–21.

Pettersen, E.F., Goddard, T.D., Huang, C.C., Couch, G.S., Greenblatt, D.M., Meng, E.C., and Ferrin, T.E. (2004). UCSF Chimera: A visualization system for exploratory research and analysis. J. Comput. Chem. *25*, 1605–1612.

Sharma, A., Mariappan, M., Appathurai, S., and Hegde, R.S. (2010). In vitro dissection of protein translocation into the mammalian endoplasmic reticulum. Methods Mol. Biol. *619*, 339–363.

Stefanovic, S., and Hegde, R.S. (2007). Identification of a targeting factor for posttranslational membrane protein insertion into the ER. Cell *128*, 1147–1159.
